# PSMD14-mediated deubiquitination of CARM1 facilitates the proliferation and metastasis of hepatocellular carcinoma by inducing the transcriptional activation of FERMT1

**DOI:** 10.1038/s41419-025-07416-3

**Published:** 2025-02-27

**Authors:** Jing Lu, Huita Wu, Ping Zhan, Yuyan Lu, Qinliang Fang, Changhong Luo, Fuqiang Wang, Jing Wen, Chengrong Xie, Zhenyu Yin

**Affiliations:** 1 Xiamen Translational Medical Key Laboratory of Digestive System Tumor, Fujian Provincial Key Laboratory of Chronic Liver Disease and Hepatocellular Carcinoma, Zhongshan Hospital of Xiamen University, School of Medicine, Xiamen University, Xiamen, Fujian Province China; 2 Department of Breast Surgery, General Surgery, Qilu Hospital, Cheeloo College of Medicine, Shandong University, Jinan, Shandong Province China; 3 Department of Oncology, Zhongshan Hospital of Xiamen University, School of Medicine, Xiamen University, Xiamen, Fujian China; 4 Department of Hepatobiliary Surgery, Xiamen Key Laboratory of Liver Diseases, Xiamen Hospital of Traditional Chinese Medicine, Beijing University of Chinese Medicine, Xiamen, Fujian Province China

**Keywords:** Cancer, Post-translational modifications

## Abstract

Hepatocellular carcinoma (HCC) is a highly potent malignancy. The enzyme coactivator-associated arginine methyltransferase 1 (CARM1) is highly expressed in different types of cancer. However, the precise levels of expression, clinical significance, biological functions, and molecular mechanisms of CARM1 in HCC, particularly related to the downstream genes regulated by CARM1 through histone arginine methylation, remain unclear. In this study, we presented findings from the TCGA database and clinical samples, which collectively demonstrated the overexpression of CARM1 in HCC. Additionally, we found that the upregulation of CARM1 was mediated by PSMD14-induced deubiquitination. CARM1 promoted the proliferation and metastasis of HCC cells in vitro and in vivo. Mechanistic investigations further revealed that FERMT1 is a downstream gene of CARM1, and CARM1 activates the transcription of FERMT1 through the dimethylation of arginine 17 on histone 3 (H3R17me2). Additionally, administering SGC2085, a CARM1 inhibitor, effectively suppressed the malignant behaviors of HCC cells. To summarize, our findings provided strong evidence that CARM1 can serve as a key oncoprotein; thus, it holds promise as a therapeutic target for HCC.

## Introduction

Coactivator-associated arginine methyltransferase 1 (CARM1), also known as protein arginine methyltransferase 4 (PRMT4), was originally identified as a protein that binds to the p160 steroid receptor coactivator GRIP1 [1]. CARM1 has an N-terminal pleckstrin homology-like domain (PH-like), a C-terminal transactivation domain, and a central catalytic domain containing four conserved PRMT motifs [2]. It regulates various cellular processes, including transcriptional regulation, pre-mRNA splicing, the cell cycle, and DNA damage repair [3–7]. CARM1 methylates arginine 17 (H3R17) and H3R26 of histone 3 and also forms transcriptional complexes with RNA polymerase II complex component 1 (PAF1), androgen receptor (AR), estrogen receptor (ER), and histone acetyltransferase P300 to activate downstream gene expression [8–10]. CARM1 also methylates non-histone proteins, regulating their functions. For example, CARM1 activates aerobic glycolysis and promotes tumor development by methylating the key glycolytic enzyme PKM2 [11]. It facilitates the growth and metastasis of breast cancer by methylating arginine 1064 of the chromatin remodeling factor BAF155 [12]. Additionally, CARM1 enhances sensitivity to chemotherapeutic drugs in breast cancer cells by methylating MED12 [13]. However, the expression level, clinical significance, biological function, and molecular mechanism of CARM1 in hepatocellular carcinoma (HCC), particularly the downstream genes regulated by CARM1 through histone arginine methylation, remain unclear.

Studies have found that CARM1 can be regulated by post-translational modifications. CARM1 is phosphorylated on several residues, such as T131, which is phosphorylated by glycogen synthase kinase 3 beta to prevent proteasomal degradation [14]. Some post-translational modifications of CARM1 are important [15]. For example, phosphorylation of serine 217 of CARM1 inhibits the methylation activity of PRMT4 [16, 17]; O-acetylglycosylation modulates the substrate specificity of CARM1 [18]. CARM1 undergoes methylation, affecting its transcriptional activation and pre-mRNA splicing functions [15]; nuclear AMPK regulates the ubiquitination-mediated degradation of CARM1 through the E3 ligase SKP2 [19]. The ubiquitin-proteasome system is one of the main pathways for degrading proteins. Under the sequential actions conducted by several enzymes, including activating (E1), conjugating (E2), and ligating (E3) enzymes, ubiquitin (Ub) is attached to the lysine residue of substrate proteins through monoubiquitination and polyubiquitination [20]. Ubiquitination can be reversed by deubiquitinating enzymes (DUBs), and dysregulation of DUBs can cause various diseases, including cancer [21]. The deubiquitinase PSMD14, the 26S proteasome non-ATPase regulatory subunit 1, belongs to the JAMM domain protease family [22]. High expression levels of PSMD14 also predict poor prognosis in various types of cancer [22, 23]. PSMD14 plays important roles in various biological processes, such as proliferation, metastasis, DNA damage response, immune tolerance, and multidrug resistance [22–25]. In HCC, PSMD14 stabilizes E2F1, which upregulates the expression of survivin and FOXM1, thereby facilitating the growth of HCC [26]. Additionally, PSMD14 deubiquitinates the TGF-β receptor and caveolin-1 to facilitate HCC metastasis [27]. In another study, we revealed that PSMD14 deubiquitinates and stabilizes GRB2 to promote the growth and metastasis of HCC [28]. By conducting mass spectrometric analysis, we identified CARM1 as a potential PSMD14-interacting protein. However, the regulatory relationship between PSMD14 and CARM1 remains unclear.

In this study, we investigated the clinical significance of CARM1 upregulation in HCC tissue samples and determined the oncogenic role of CARM1 in HCC. Our findings revealed that PSMD14 interacts with CARM1, which prevents its degradation. We also found that FERMT1 serves as a downstream gene of CARM1-mediated histone methylation in HCC, influencing the proliferation and metastasis of HCC. Additionally, the CARM1 inhibitor SGC2085 strongly inhibited HCC. Our findings revealed a novel PSMD14-CARM1-FERMT1 axis that facilitates the progression of HCC.

## Materials and methods

### Tissue collection

We collected 66 pairs of HCC tissues and their adjacent normal liver tissues from the Department of Hepatobiliary Surgery, Zhongshan Hospital of Xiamen University. These tissues were obtained from patients who underwent curative hepatectomy from 2016 to 2020. The collected samples were immediately frozen with liquid nitrogen and stored at –80 °C. All patients provided informed consent and none underwent prior treatments such as chemotherapy, targeted therapy, or interventional therapy. The study was approved by the ethics committee of Zhongshan Hospital of Xiamen University.

### Cell culture

PLC/PRF/5, Huh7, SK-Hep-1, and HEK293T cells were purchased from Cellcock Company (Guangzhou China). These cell lines were compared with those in the STR database for authentication. All cell lines were cultured with high-glucose Dulbecco’s modified Eagle medium (DMEM, Gibco) supplemented with 10% fetal bovine serum (FBS, Gibco) and 1% penicillin/streptomycin (Gibco) at 37 °C in a humidified incubator with 5% CO_2_.

### Immunohistochemistry (IHC)

Tissues were fixed with 10% neutral formalin, embedded in paraffin, and cut into sections (4 μm thick) for IHC staining. Serial sections were deparaffinized, hydrated, and incubated in 3% H_2_O_2_ for 20 min at room temperature and then stained with anti-PSMD14 (1:3200, A9608, ABclonal) and anti-CARM1 (1:1000, NB200-342, Novus) at 4 °C overnight. After washing three times in phosphate-buffered saline (PBS) for 5 min each, secondary antibodies (goat anti-rabbit) were added to the slides, which were subsequently incubated for 1 h at room temperature. The sections were visualized with diaminobenzidine (DAB; Maixin Biotechnology, Fuzhou, China) and counterstained with hematoxylin (Maixin Biotechnology) for 3–5 min at room temperature. To avoid bias, two pathologists who were blinded to the clinical data examined the IHC-stained slides separately. They randomly counted 100 cells in the microscopic field at 200× magnification. All sections were classified into five ranges depending on the percentage of cells with positive staining: 0 = negative, 1 = 0–25%, 2 = 26–50%, 3 = 51–75%, and 4 = 76–100%. Moreover, we grouped all sections into four levels based on the staining intensity as follows: 0 = negative, 1 = pale yellow, 2 = medium yellow, and 3 = tawny. The final score was the product of the proportion multiplied by the intensity score; the final score ranged from 0 to 12, where 0 to 6 was considered to indicate low expression and 7 to 12 was considered to indicate high expression.

### Western blotting

Cellular protein was extracted using radioimmunoprecipitation assay (RIPA) lysis buffer (Beyotime; Beijing, China) containing a protease inhibitor (APExBIO). Proteins were quantified using a bicinchoninic acid (BCA) kit (Thermo Fisher, US) and then boiled in an SDS-PAGE loading buffer (Solabio). Next, 10% SDS-PAGE was used to separate the protein samples, which were transferred onto polyvinylidene fluoride (PVDF) membranes (Millipore). The PVDF membranes were blocked with 5% non-fat milk (Bio-Rad, US) for at least 1 h at room temperature and then incubated with primary antibodies overnight at 4 °C, followed by incubation with anti-rabbit-HRP or anti-mouse-HRP secondary antibodies. The target proteins were immediately visualized via enhanced chemiluminescence (ECL; Millipore). The following antibodies were used: anti-PSMD14 (4197 s, Cell Signaling), anti-CARM1 (NB200-342, Novus), anti-β-actin (3700, Cell Signaling), anti-FLAG (F1804, Sigma), anti-HA (3724, Cell Signaling), anti-ubiquitin (60396, Cell Signaling), anti-GFP (ab32146, Abcam), and anti-IgG (2729, Cell Signaling).

### RNA extraction and quantitative real-time PCR (qRT-PCR)

The standard TRIzol (Invitrogen, US) protocol was used for extracting total RNA, followed by reverse transcription using the One-Step gDNA Removal and cDNA Synthesis Kit (TransGen, Beijing, China). Then, qRT-PCR was performed in a Lightcycler 96 Real-Time PCR System (Roche, Switzerland) using Taq Pro Universal SYBR qPCR Master Mix (Vazyme). The results were normalized to those of GAPDH and presented as the mean of at least three independent experiments. The primers used were as follows: GAPDH-Forward: GGTGTGAACCATGAGAAGTATGA,

GAPDH-Reverse: GAGTCCTTCCACGATACCAAAG;

CARM1-Forward: GAAGGAGATTTGCACAGGATAGA,

CARM1-Reverse: GACAGCCACACGGTCATTAT;

PSMD14-Forward: CTTAGACTTGGAGGAGGTATGC,

PSMD14-Reverse: CAGTGCCAGGGAAGAGATATAG.

### Construction of stable overexpression and knockdown cells

For the purpose of CARM1 overexpression, pLVX-Puro, and pLVX-Puro-CARM1-FLAG (containing 3×FLAG tags) plasmids were purchased from Public Protein/Plasmid Library Corporation (Nanjing; Jiangsu Province, China). In order to carry out knockdown, pLV [shRNA]-mCherry-U6, pLV [shRNA]-mCherry-U6 > CARM1[shRNA], and pLV [shRNA]-mCherry-U6 > CARM1[shRNA] plasmids were purchased from VectorBuilder (Guangzhou; Guangdong Province, China). The lentiviral particles were packed in HEK293T cells following the standard protocol. The medium was collected after 48 h of transfection and added to the target cells with polybrene (8 μg/mL) for 48 h. Stable cells were selected with puromycin (Beyotime, Beijing, China). The shRNA sequences used are as follows:

shCARM1-1-Forward: gatccGCAGCCATGAAGATGTGTGTTTCAAGAGAACACACATCTTCATGGCTGTTTTTTg;

shCARM1-1-Reverse: aattcAAAAAACAGCCATGAAGATGTGTGTTCTCTTGAAACACACATCTTCATGGCTGCg;

shCARM1-2-Forward: gatccGTGGCCAAGTCTGTCAAGTATTCAAGAGATACTTGACAGACTTGGCCATTTTTTg;

shCARM1-2-Reverse: aattcAAAAAATGGCCAAGTCTGTCAAGTATCTCTTGAATACTTGACAGACTTGGCCACg.

### Transwell assay

The migration and invasion abilities of cells were detected by Transwell assays using 24-well transwell chambers (8 μm pore size; Corning Incorporated; New York, NY, US) coated with or without Matrigel (BD bioscience). The cells were mixed with 200 μL of serum-free DMEM and seeded in the upper chambers, and the lower layer was supplemented with 200 μL of DMEM supplemented with 10% FBS. After incubation, 500 μL of 4% paraformaldehyde was used to fix the cells on the bottom surface of the chambers for 30 min at room temperature. These cells were subsequently stained with 1% crystal violet for 30 min at room temperature. Finally, 10 random fields were selected for imaging and quantification under a microscope.

### Cell proliferation assay

To detect the proliferation of HCC cells, cell counting kit-8 (CCK-8) assays (Dojindo; Beijing; China) were performed. HCC cells (2000 cells per well) were seeded in 96-well plates in 100 μL of medium. The absorbance was measured at 450 nm after the cells were incubated with 10 μL of CCK-8 solution at 37 °C for 1.5 h. The CCK-8 assay for each cell line was independently repeated three times.

### Immunofluorescence (IF) staining assay

The cells were seeded on glass coverslips in six-well plates, fixed with 1% formaldehyde for 10 min, and permeabilized with 0.2% Triton X-100 in PBS for 10 min at room temperature. The cells were washed twice with PBS, and 5% BSA in PBS was used to block them for 1 h. Then, the cells were incubated with primary antibodies at 4 °C overnight, washed three times in PBS, and incubated with secondary antibodies for 1 h at room temperature. The cell nuclei were counterstained with 500 nM DAPI (Sigma). Finally, photographs were taken using a confocal microscope (Zeiss).

### Coimmunoprecipitation (co-IP) assay

The cells were collected and lysed with IP lysis buffer (Beyotime, Beijing, China). After extracting total proteins, Dynabeads Protein G (Invitrogen; Beijing; China) and the appropriate antibodies were added to the cell lysis buffer, and the incubation was prolonged overnight at 4 °C on a rocking platform. After the beads were washed thrice, the proteins immunoprecipitated with a loading buffer were boiled and evaluated by Western blotting analysis.

### Chromatin immunoprecipitation assay (ChIP) and high-throughput sequencing

A ChIP assay was performed following the instructions provided by the manufacturer using a Magna ChIP™ A/G Chromatin Immunoprecipitation Kit (Millipore). Briefly, the cells were cross-linked in a 1% formaldehyde solution at room temperature for 10 min. To terminate the cross-linking process, glycine was added, and the mixture was incubated for 5 min. The samples were washed with ice-cold PBS, scraped, and resuspended in PBS. After washing, the cell pellets were lysed with a cell lysis buffer. The lysates were incubated on ice for 10 min and centrifuged at 5000 rpm for 5 min at 4 °C to pellet the nuclei. The pellets formed were lysed using a nuclear lysis buffer containing 50 mM Tris-HCl (pH 8.0), 10 mM EDTA, 1% SDS, 1 mM PMSF, and a proteinase inhibitor cocktail. Then, the lysates were diluted with an IP dilution buffer. The nuclear lysates were sonicated, and the debris was removed through centrifugation. For immunoprecipitation, CARM1 antibodies (Novus), H3R17me2 antibodies (Active Motif), or IgG antibodies (Millipore) were mixed with the nuclear lysates. The coprecipitated DNA was purified, and the expression levels of the target genes were quantified by qRT-PCR. The sequences of the primers used were as follows:

FERMT1-A-Forward: ATTAGCCAGGTGTGGTGGCAC,

FERMT1-A-Reverse: GGAGTGCAATGGTGTGATCTC;

FERMT1-B-Forward: GCTAGTCTTGAACTCCTGACC,

FERMT1-B-Reverse: GACCGTGGGACTAAAAATGAA;

FERMT1-C-Forward: GTACGGTCCTGTTGTGTCCTC,

FERMT1-C-Reverse: ATCTTGGCGCTGTGAGGAGAG;

FERMT1-D-Forward: AGTGCACCGGGCACGCCGCGC,

FERMT1-D-Reverse: GCTCAGCTAAGGCTGCGGCAG.

High-throughput DNA sequencing libraries were prepared using the VAHTS Universal DNA Library Prep Kit for Illumina V3 (Catalog No. ND607, Vazyme). Library fragments in the 200-500 base pair range were enriched, quantified, and sequenced using the Novaseq 6000 sequencer (Illumina) with the PE150 protocol. Data analysis was conducted by SeqHealth Technology Co., Ltd. (Wuhan, China).

### RNA sequencing

Total RNA was extracted from shCON and shCARM1 Huh7 cells using TRIzol Reagent (Invitrogen, cat. no. 15596026) according to the method described by Chomczynski et al. (DOI: 10.1006/abio.1987.9999). DNA digestion was performed post-RNA extraction using DNase I. RNA quality was assessed by measuring the A260/A280 ratio with a Nanodrop™ OneC spectrophotometer (Thermo Fisher Scientific Inc.). RNA integrity was further confirmed by 1.5% agarose gel electrophoresis. The quantity of qualified RNA was determined using a Qubit 3.0 fluorometer and the Qubit™ RNA Broad Range Assay Kit (Life Technologies, Q10210). For RNA sequencing, 2 μg of total RNA was used for library preparation with the KCTM Stranded mRNA Library Prep Kit for Illumina® (Catalog No. DR08402, Wuhan Seqhealth Co., Ltd., China) according to the manufacturer’s instructions. PCR products corresponding to 200–500 bp were enriched, quantified, and sequenced on a NovaSeq 6000 sequencer (Illumina) using the PE150 protocol. RNA-Seq data analysis was performed by Seqhealth Technology Co., Ltd. (Wuhan, China).

### In vivo deubiquitination assay

HA-ubiquitinated FLAG-CARM1 or endogenous CARM1 was immunoprecipitated by FLAG antibodies (Sigma) or CARM1 antibodies (Novus), respectively, under denaturing conditions (1% SDS, 50 mM Tris-HCl, pH 7.5, 150 mM NaCl, 1 mM EDTA) and incubated at 95 °C for 5 minutes. Subsequently, the FLAG-CARM1 and CARM1 proteins were purified by anti-FLAG M2 magnetic beads (Sigma-Aldrich) and anti-CARM1 antibodies (Novus) conjugated with Protein G magnetic beads (Thermo Fisher), respectively. Then incubated at 4 °C overnight with gentle rotation to allow specific binding. The eluted proteins were subjected to immunoblotting with antibodies specific to HA or ubiquitin.

### In vitro deubiquitination assay

HEK293T cells expressing HA-Ubi-FLAG-CARM1 were lysed in lysis buffer (50 mM Tris-HCl, pH 7.5, 150 mM NaCl, 1% NP-40, 1 mM EDTA, and protease inhibitor cocktail). HA-Ubi-FLAG-CARM1 was immunoprecipitated using protein G-agarose beads (Thermo Fisher Scientific) pre-incubated with anti-FLAG antibody (Sigma-Aldrich) at 4 °C for 4 h with gentle rotation. Similarly, GFP-tagged wild-type (WT) or mutant PSMD14 protein complexes were isolated from HEK293T cells expressing the respective constructs. Cell lysates were prepared and incubated with protein G-agarose beads pre-bound to an anti-GFP antibody (Thermo Fisher) under the same conditions as FLAG-CARM1. Next, the protein complexes were mixed in a reaction buffer containing 50 mM Tris (pH 7.5), 10 mM MgCl_2_, 1 mM dithiothreitol (DTT), 100 mM NaCl, and 1 mM ATP. The reaction mixture was incubated at 37 °C for 1 h with gentle agitation. Following incubation, FLAG-CARM1 was re-purified using protein G-agarose beads pre-bound with anti-FLAG antibody. Subsequently, SDS-PAGE and immunoblotting were performed.

### Xenograft mouse models

A total of 2 × 10^6^ cells with or without CARM1 knockdown were injected subcutaneously into the backs of nude mice (6-week-old male BALB/c athymic nude mice). After 1 week, the volume of the tumor was measured using the formula 1/2 × length × width^2^ every 3 days with Vernier calipers. After 28 days, the tumors were harvested, weighed, and used for further analysis. To inject SGC2085, when the tumors were about 200 mm^3^, the mice were randomly divided into two groups (*n* = 4 mice per group). Then, SGC2085 (10 mg/kg, diluted in sterile water containing 30% PEG300, 5% Tween-80, and 10% DMSO) or an equal volume of solvent was injected intraperitoneally every four days. After six injections, the tumors were harvested and weighed. All animal experiments were approved by the Animal Care and Use Committee of Xiamen University.

### Statistical analysis

All statistical analyses were performed using SPSS 20 (SPSS, Inc., Chicago, IL). The data were presented as the mean ± standard deviation (SD), and three independent replicates were performed. The differences between groups were determined by conducting Student’s *t*-tests or ANOVA. The Chi-square test was conducted to analyze the relationships between CARM1 expression and clinical features. The Kaplan-Meier survival test and log-rank test were performed to analyze the relationship between the expression of PSMD14 or CARM1 and the prognosis of HCC patients. All differences among and between groups were considered to be statistically significant at *P* < 0.05.

## Results

### Overexpression of CARM1 indicates poor clinical outcomes in HCC patients

**Fig. 1:**
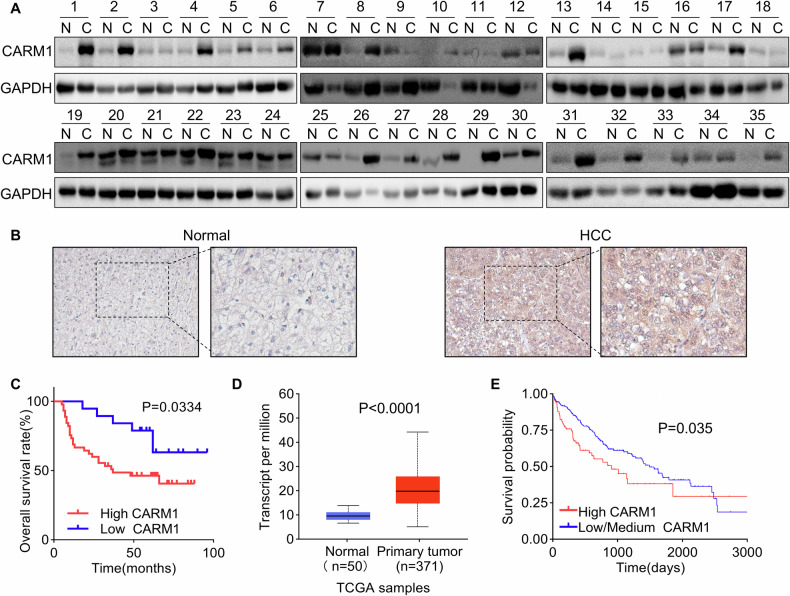
CARM1 is upregulated in HCC tissues and indicates poor clinical outcomes. **A** Western blotting analysis showed the expression of CARM1 in 35 pairs of HCC and adjacent normal liver tissues. **B** The expression of CARM1 in 66 pairs of HCC and corresponding adjacent normal liver tissues was examined via an IHC assay. **C** Kaplan-Meier survival analysis of overall survival in HCC patients stratified by CARM1 expression. Patients with low expression (*n* = 19) had lower expression values in HCC tissues than in normal tissues, while patients with high expression (*n* = 47) had higher expression values in HCC tissues than in normal tissues. **D** CARM1 mRNA levels in normal and primary HCC tumor tissues from the TCGA database. **E** Kaplan-Meier survival analysis of HCC patients from the TCGA database was performed according to CARM1 mRNA expression. Patients with high CARM1 expression had expression values in the >3rd quantile, while patients with low CARM1 expression had expression values in the <3rd quartile.

A study reported that CARM1, an oncogene, is dysregulated in several types of cancer [29]. However, the expression pattern of CARM1 and its clinical significance in HCC tissues remain elusive. First, we determined the differential expression of CARM1 in 35 paired HCC and corresponding adjacent normal tissues. The results of Western blotting assays showed that the expression level of CARM1 was significantly greater in HCC tissues than in corresponding adjacent normal tissues (Fig. 1A). For further validation, IHC staining of CARM1 was performed in 66 HCC tissues and paired adjacent normal tissues. CARM1 was upregulated in 71.2% (47/66) of HCC tissues compared to its expression in adjacent normal tissues (Fig. 1B).

To determine the clinical significance of CARM1 overexpression in HCC patients, a correlation analysis between CARM1 expression and clinicopathological features was performed, and the results showed that CARM1 expression was significantly related to tumor size (*P* = 0.0443), the level of differentiation (*P* = 0.0239), and satellite foci (*P* = 0.0272). However, CARM1 expression was not associated with other factors, including age, sex, AFP level, PVTT, HBV DNA level, or liver cirrhosis (Table 1). Moreover, the results of the Kaplan-Meier survival analysis revealed that patients with high expression of CARM1 in HCC had a lower overall rate than those with low CARM1 expression (Fig. 1C). Additionally, the UALCAN online tool (http://ualcan.path.uab.edu/index.html) and TIMER 2.0 were used to validate the reliability of our results. Consistent with our findings, the CARM1 expression levels were higher in primary HCC tissues (Fig. 1D and Supplementary Fig. 1A). A higher tumor grade and advanced cancer stage were associated with higher expression of CARM1 (Supplementary Fig. 1B, C). The Kaplan-Meier analysis of the data in the TCGA database using the Kaplan-Meier plotter online tool (https://ualcan.path.uab.edu/cgi-bin/TCGA-survival) showed that patients with high CARM1 expression exhibited markedly shorter overall survival than those with low CARM1 expression (*P* = 0.035) (Fig. 1E). The data from our cohort and the TCGA database indicated that CARM1 is upregulated in HCC tissues and associated with the malignant progression of HCC.

**Table 1:** Correlations between CARM1 expression and the clinicopathological features of patients with HCC.

Clinicopathological factors	CARM1 expression		*X*^2^	*P* value
	High	Low		
Age				
<60	27	12	0.1826	0.6692
≥60	20	7		
Gender				
Male	38	13	1.19	0.2753
Female	9	6		
Tumor size				
≤5 cm	12	9	4.047	0.0443*
>5 cm	35	10		
Differentiation level				
Low/Medium	47	17	5.102	0.0239*
High	0	2		
Satellite foci				
Without	26	16	4.881	0.0272*
With	21	3		
AFP (μg/L)				
<400	29	14	0.8556	0.3550
≥400	18	5		
PVTT				
Without	22	11	0.6652	0.4147
With	25	8		
HBV DNA				
<1000	26	13	0.9608	0.3270
≥1000	21	6		
Liver cirrhosis				
Without	37	13	0.7819	0.3765
With	10	6		
With	20	5		

*AFP* alpha-fetoprotein, *PVTT* portal vein tumor thrombus, *HBV* hepatitis B virus. (*p < 0.05)

### PSMD14 interacts with and stabilizes CARM1

**Fig. 2:**
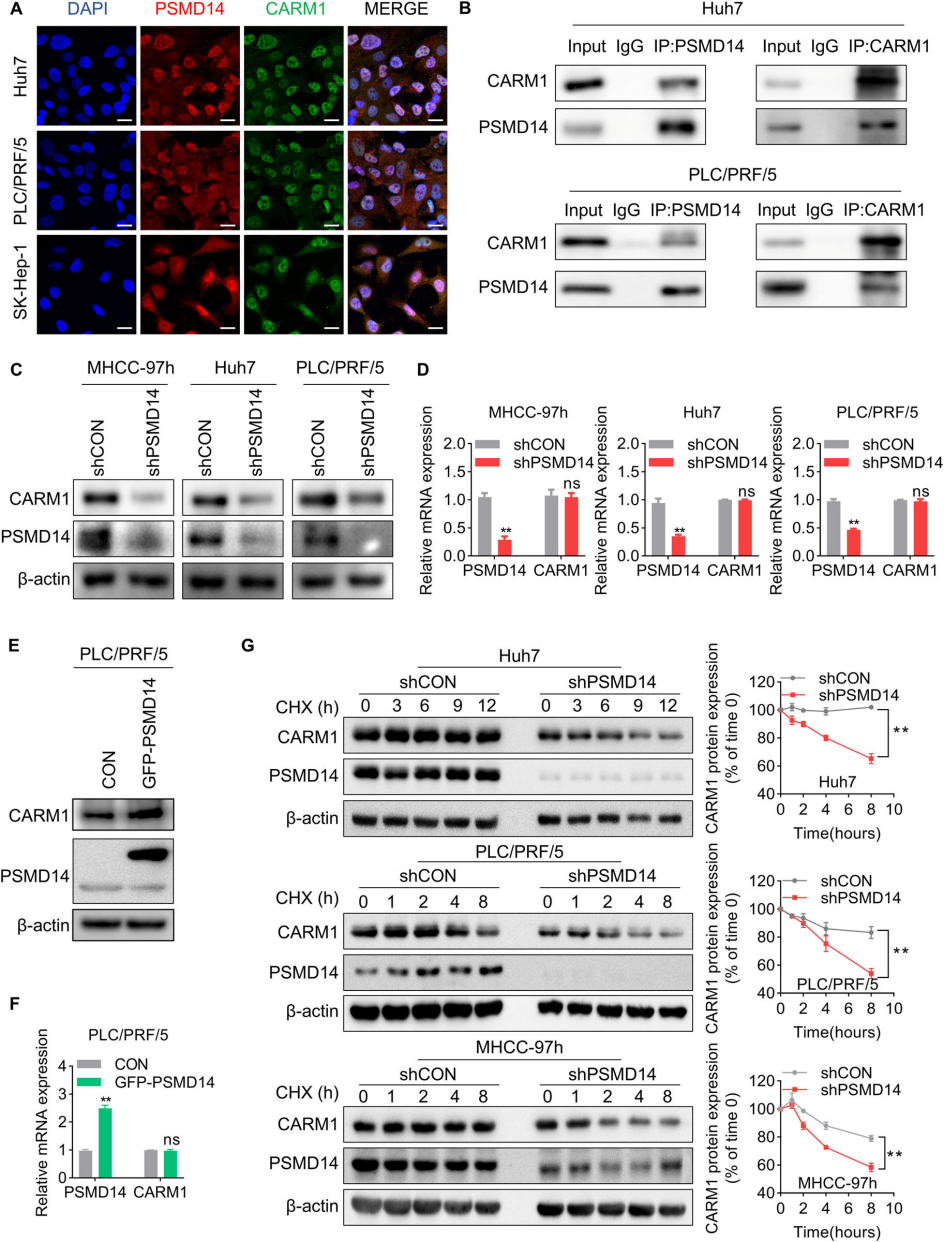
The stability of CARM1 is regulated by PSMD14. **A** Representative confocal microscopy image of IF colocalization analysis of CARM1 and PSMD14 in HCC cell lines. Scale bar, 20 μm. **B** Endogenous PSMD14 proteins were immunoprecipitated with anti-PSMD14 antibodies and then analyzed by immunoblotting (left panel). Endogenous CARM1 proteins were immunoprecipitated with anti-CARM1 antibodies and then analyzed by immunoblotting (right panel). The IgG antibody was used as the control. **C** Western blotting assays showed the expression of CARM1 in control and PSMD14-knockdown HCC cells. **D** CARM1 mRNA expression levels in control and PSMD14-knockdown HCC cells were determined by qRT-PCR. **E** The protein expression levels of CARM1 were assessed by Western blotting analysis in HCC cells with either empty vector or PSMD14 overexpression. **F** The mRNA expression levels of CARM1 were assessed by qRT-PCR in HCC cells with either control or PSMD14 overexpression. **G** Control and PSMD14-knockdown HCC cells were treated with CHX (20 µM) for the indicated times, and endogenous CARM1 protein expression was detected by Western blotting analysis (left). The expression levels of CARM1 were determined by densitometry. The level of CARM1 protein in CHX-untreated cells (0 h) was set to 100% (*n* = 3; *p < 0.05 and **p < 0.01).

Next, we investigated the regulatory mechanism of the overexpression of CARM1 in HCC. By conducting mass spectrometry analysis, in our previous study, we identified 710 proteins potentially associated with the deubiquitinase PSMD14 [28]. CARM1 was identified as one of the main PSMD14-interacting proteins, indicating that PSMD14 might regulate CARM1. Through double IF labeling for CARM1 and PSMD14, we found that CARM1 and PSMD14 were co-localized (Fig. 2A). The interaction between endogenous CARM1 and PSMD14 was confirmed via co-IP experiments (Fig. 2B).

As PSMD14 functions as a deubiquitinase to modulate protein stability, we examined whether PSMD14 can regulate CARM1 through this mechanism. Decreasing PSMD14 expression did not affect the expression level of CARM1 mRNA; however, the results of Western blotting analyses showed that PSMD14 knockdown resulted in a substantial decrease in CARM1 protein levels in HCC cells (Fig. 2C, D). In contrast, the overexpression of PSMD14 upregulated the expression of the CARM1 protein but did not increase its mRNA level (Fig. 2E, F). To determine whether CARM1 is stabilized by PSMD14, we conducted cycloheximide (CHX) pulse-chase assays to measure the degradation rates of endogenous CARM1 under the effect of PSMD14 knockdown in HCC cells. CARM1 degradation was strongly accelerated in PSMD14-knockdown cells compared to that in control cells (Fig. 2G). These results suggested that PSMD14 interacts with CARM1 and prevents its degradation.

### PSMD14 deubiquitinates CARM1

**Fig. 3:**
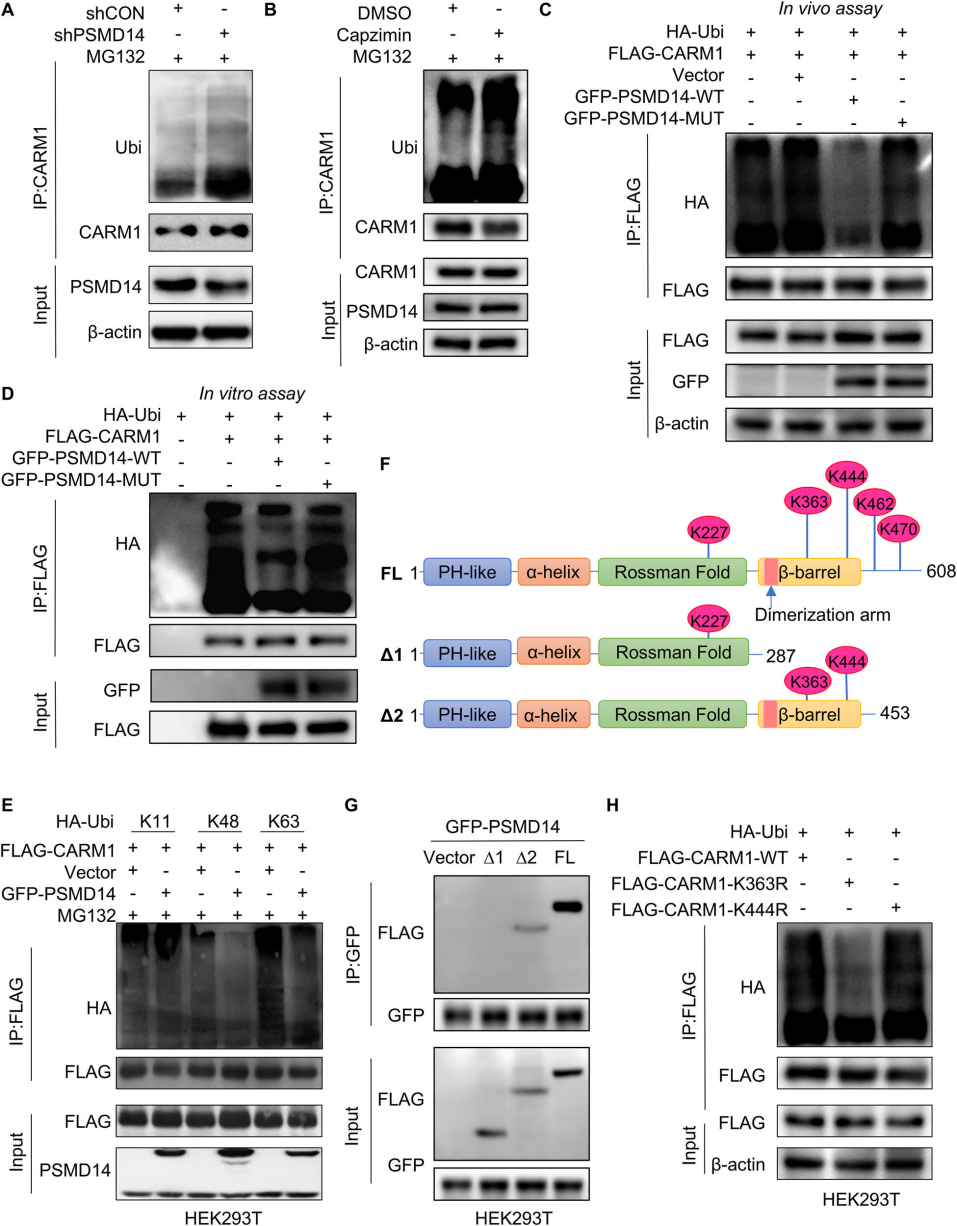
PSMD14 deubiquitinates CARM1. **A** Control and PSMD14-knockdown Huh7 cells were treated with MG132 at 10 μM for 6 h. The cell lysates were immunoprecipitated with an anti-CARM1 antibody, and the immunocomplexes were immunoblotted with anti-CARM1 and anti-ubiquitin antibodies. **B** Huh7 cells were pretreated with 10 μM capzimin for 24 h, and cells treated with equal amounts of DMSO served as controls. All cells were treated with MG132 at 10 μM for 6 h. The cell lysates were then immunoprecipitated with anti-CARM1 antibodies, and the immunocomplexes were immunoblotted with anti-CARM1 and anti-ubiquitin antibodies. **C** FLAG-CARM1 and HA-Ubi were transiently transferred into HEK293T cells expressing GFP-PSMD14-WT or GFP-PSMD14-MUT. Cells were then treated with MG132 (10 μM) for 6 h. The cell lysates were immunoprecipitated with an anti-FLAG M2 affinity gel. The ubiquitination levels of CARM1 were detected using anti-HA antibodies. **D** FLAG-CARM1 and HA-Ubi were transiently transferred into HEK293T cells, which were subsequently purified with FLAG antibodies and protein G beads. Moreover, GFP-PSMD14-WT and GFP-PSMD14-MUT were separately transferred into another two sets of HEK293T cells and purified with GFP antibodies and protein G beads. The purified Ubi-FLAG-CARM1 and GFP-PSMD14 proteins were incubated for 1 h. Then, FLAG-CARM1 was immunoprecipitated with anti-FLAG M2 affinity beads and detected with the indicated antibodies. **E** HEK293T cells were transfected with the indicated plasmids and treated with MG132 (10 μM) for 6 h. The cell lysates were immunoprecipitated with anti-FLAG M2 affinity beads. The ubiquitination levels of CARM1 were detected using anti-HA antibodies. **F** Schematic illustration of CARM1 truncation plasmids and lysine sites predicted to be modified by ubiquitination. **G** HEK293T cells expressing GFP-PSMD14 were transfected with FLAG-tagged full-length or truncated CARM1. The cell lysates were immunoprecipitated with anti-GFP antibodies. **H** HEK293T cells were transfected with the indicated plasmids and treated with MG132 (10 μM) for 6 h. The cell lysates were immunoprecipitated with an anti-FLAG M2 affinity gel. The ubiquitination levels of CARM1 were detected using anti-HA antibodies.

Next, the mechanism of PSMD14-mediated stability of CARM1 was investigated. To determine whether PSMD14 stabilizes CARM1 via deubiquitination, we performed an in vivo deubiquitination assay to test the level of CARM1 ubiquitination in control and PSMD14-knockdown Huh7 cells. The level of ubiquitinated CARM1 increased substantially after the depletion of PSMD14 (Fig. 3A). Similarly, Capzimin, a specific inhibitor of PSMD14 [30], also led to a sharp increase in the CARM1 ubiquitination level (Fig. 3B), further suggesting that PSMD14 deubiquitinated CARM1. The JAMM domain of PSMD14 plays a key role in maintaining its deubiquitinating enzymatic activity [31]. We constructed a plasmid expressing a GFP-tagged PSMD14 mutant with deletion of the JAMM motif (named GFP-PSMD14-MUT). Compared to wild-type PSMD14, mutant PSMD14 failed to decrease the ubiquitination level of exogenous CARM1 (Fig. 3C). By conducting an in vitro deubiquitination assay, we also found that PSMD14 directly deubiquitinated CARM1. Only purified GFP-PSMD14-WT but not GFP-PSMD14-MUT deubiquitinated CARM1 in vitro (Fig. 3D). These results indicated the indispensable role of the deubiquitinating activity of PSMD14 in the regulation of CARM1.

Next, we investigated the polyubiquitin modifications on the CARM1 protein that are specifically influenced by deubiquitination mediated by PSMD14. FLAG-CARM1, GFP-PSMD14, and various ubiquitins (WT, K11-, K48-, or K63-only ubiquitin-HA) were separately transfected into HEK293T cells. After transfecting individual plasmids into cells, we found that ectopic PSMD14 substantially decreased K48-linked and K63-linked ubiquitin on CARM1 but did not affect K11-linked ubiquitin on CARM1 (Fig. 3E).

To elucidate the underlying mechanism by which PSMD14 deubiquitinates CARM1, we used UbPred, an online tool for predicting the ubiquitination sites of substrates. Five potential lysine ubiquitination sites on human CARM1 (K227, K363, K444, K462, and K470) were predicted. Based on the predicted ubiquitination sites, we constructed a full-length CARM1 plasmid and two C-terminal truncated CARM1 plasmids (1–287 aa and 1–453 aa) with N-terminal FLAG tags (Fig. 3F). We then transiently transfected the GFP-PSMD14 plasmid along with the above three derived CARM1 plasmids separately into HEK293T cells. The resulting proteins were coimmunoprecipitated with anti-GFP antibodies and immunoblotted with anti-FLAG antibodies. FL and Δ2, but not Δ1, were pulled down by GFP-PSMD14, indicating that the ubiquitination sites of CARM1 might reside at K363 and/or K444 (Fig. 3G). Hence, we mutated C-proximal K363 and K444 to arginine separately in FLAG-tagged CARM1 plasmids and transiently transfected them into HEK293T cells. After immunoprecipitation with an anti-FLAG antibody, compared to wild-type CARM1, FLAG-CARM1^K363R^, but not FLAG-CARM1^K444R^, reduced the ubiquitination of CARM1 (Fig. 3H). These results indicated that PSMD14 deubiquitinates and stabilizes CARM1 and that the residue K363 is the only site implicated in the PSMD14-mediated ubiquitination of CARM1.

### CARM1 protein expression is positively correlated with PSMD14 in HCC tissues

**Fig. 4:**
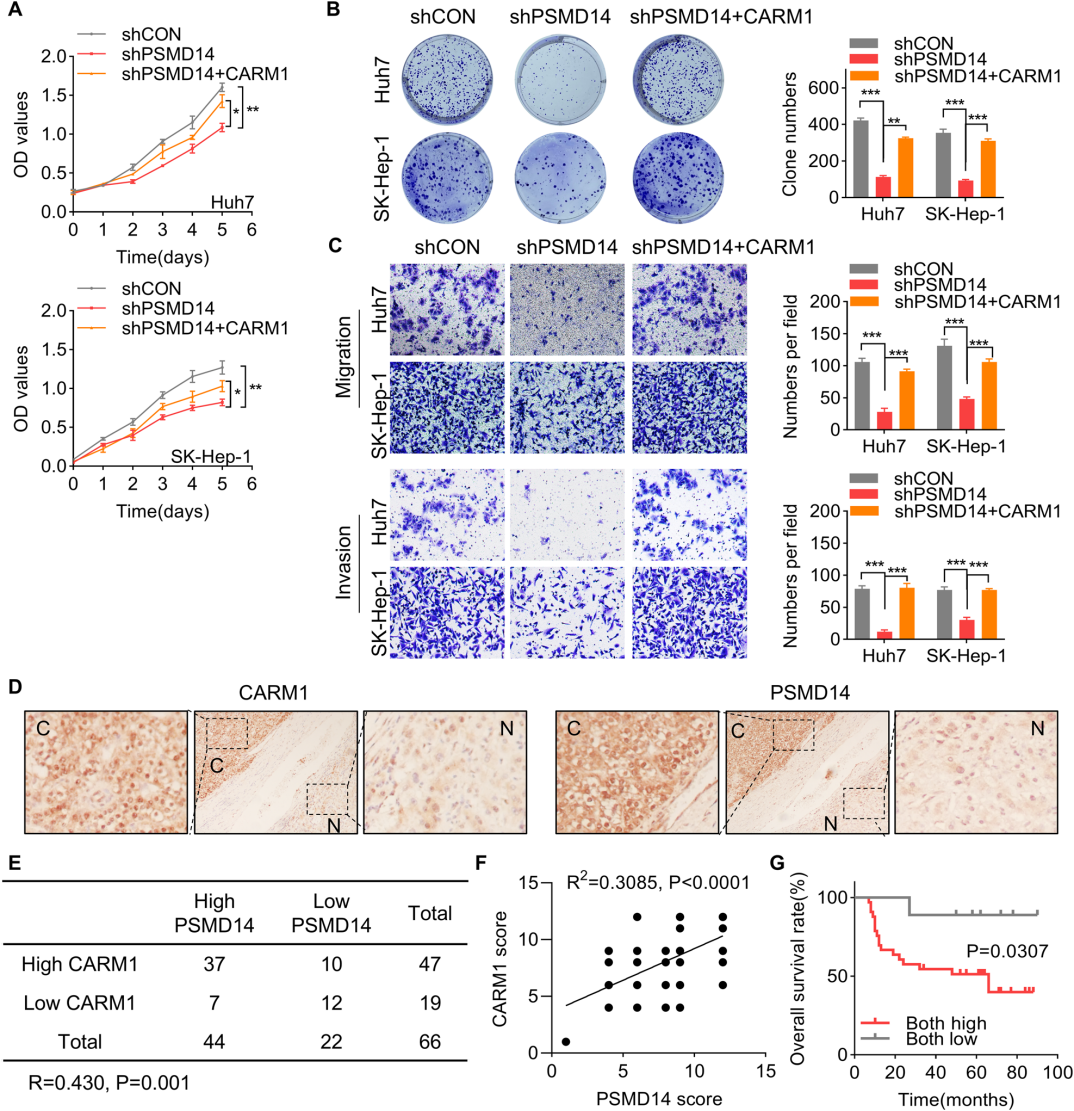
CARM1 is a downstream effector of PSMD14 that affects the prognosis of HCC patients. **A** CARM1 was transfected into HCC cells with PSMD14 knockdown. A CCK-8 assay was performed to detect proliferation. **B** CARM1 was transfected into HCC cells with PSMD14 knockdown. Then, a colony formation assay was conducted. **C** CARM1 was transfected into HCC cells with PSMD14 knockdown. Then, a transwell assay was performed to detect migration and invasion. Representative images of the transwell assay are shown. The cells in five randomly selected fields were counted under a microscope. **D** Representative images of immunohistochemical staining of PSMD14 and CARM1 in the same HCC and corresponding adjacent normal liver tissues are shown. **E** Correlation analysis of PSMD14 and CARM1 in HCC tissues. The data were statistically analyzed by the Chi-square test. R indicates the Pearson correlation coefficient. **F** Scatter diagram showing a positive correlation between PSMD14 and CARM1 in HCC tissues by IHC. **G** Survival analysis of HCC patients was conducted using Kaplan-Meier plots and log-rank tests. The patients were categorized into high and low PSMD14 and CARM1 expression groups based on IHC staining. (*n* = 3; *p < 0.05, **p < 0.01, and ***p < 0.001).

Our previous study revealed an oncogenic role of PSMD14 in HCC [28]. In this study, we examined whether CARM1 participates in this process. CARM1 was ectopically expressed following PSMD14 silencing in Huh7 and SK-Hep-1 cells. The results of the CCK8 assays revealed that CARM1 overexpression compensated for the inhibition of proliferation caused by knocking down PSMD14 (Fig. 4A). Similarly, colony formation assays revealed that restoring CARM1 expression considerably reversed the impaired colony formation effects induced by PSMD14 knockdown in HCC cells (Fig. 4B). The results of the transwell assays revealed that the ability of silencing PSMD14 to suppress migration and invasion was restored through the overexpression of CARM1 (Fig. 4C). Thus, these findings suggested that CARM1 acts as a downstream effector of PSMD14 to promote the proliferation, migration, and invasion of HCC.

Based on our finding that PSMD14 stabilizes CARM1 expression in HCC cells, we further analyzed the pathologic correlation of PSMD14 with CARM1 expression in HCC tissues. IHC staining of PSMD14 and CARM1 in the same 66 HCC and matched adjacent normal tissues was performed. In HCC tissues with elevated PSMD14 expression, high expression of CARM1 was recorded. In contrast, in HCC with a decrease in PSMD14 expression, a corresponding decrease in CARM1 expression was recorded (Fig. 4D, E, *P* = 0.001, *R* = 0.430). IHC staining for PSMD14 and CARM1 was scored based on intensity, and a positive correlation was found between PSMD14 and CARM1 expression in the same HCC tissues (Fig. 4F, *R* = 0.4283, *P* = 0.0003). Additionally, the group characterized by high levels of PSMD14 and CARM1 presented a more unfavorable prognosis than the group characterized by low expression of PSMD14 and CARM1 (Fig. 4G). Our results confirmed the clinical relevance of PSMD14-mediated regulation of CARM1 in HCC, suggesting that the upregulation of CARM1 and PSMD14 may hold promise as prognostic indicators for patients with HCC.

### CARM1 promotes the growth of HCC

**Fig. 5:**
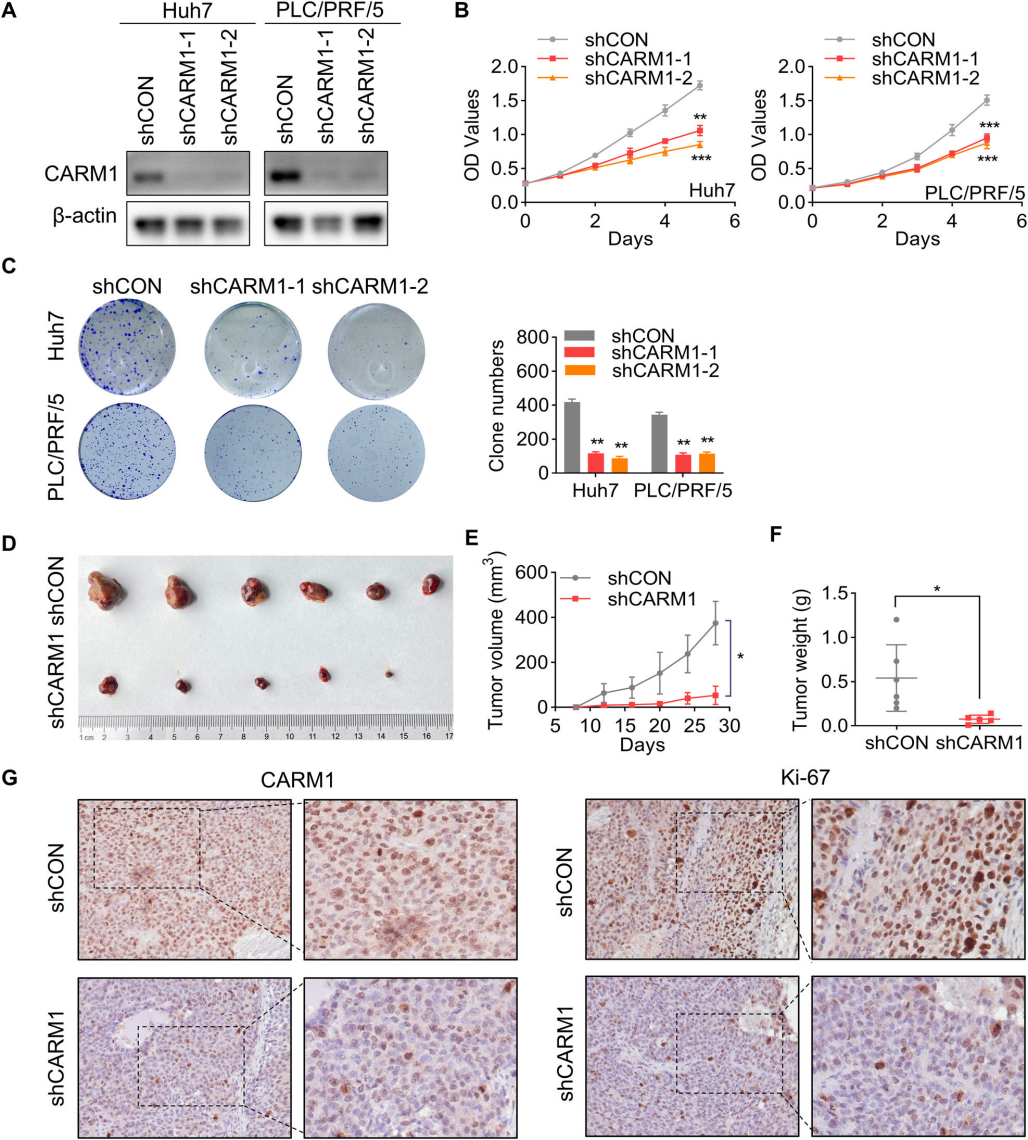
CARM1 promotes the proliferation of HCC cells. **A** Western blotting analysis showed the knockdown efficacy of CARM1 in Huh7 and PLC/PRF/5 cells infected with lentiviral particles expressing shRNAs targeting CARM1. **B** Proliferation of control and CARM1-knockdown Huh7 cells was detected by CCK-8 assays on the indicated days. **C** Colony formation assays were performed to detect the proliferation of control and CARM1-knockdown HCC cells. The data are presented in a bar chart. **D** Control or CARM1-knockdown Huh7 cells were subcutaneously injected into nude mice for observation of tumor growth. **E** The tumor volume was measured every three days and is presented as a line graph. **F** The tumor weights of the xenografts from the different groups were calculated. **G** Immunohistochemical analysis of mouse subcutaneous tumors was performed with anti-CARM1 and anti-Ki-67 antibodies. (*n* = 3; *p < 0.05, **p < 0.01, and ***p < 0.001).

To determine the functional significance of CARM1 in HCC, we generated stable cell lines with CARM1 knockdown using Huh7 and PLC/PRF/5 cells (Fig. 5A). We conducted CCK8 and colony formation assays to evaluate the effect of CARM1 knockdown on cell proliferation. Compared to the proliferation of the control cells, the proliferation of the CARM1-knockdown cells was strongly inhibited (Fig. 5B, C), whereas CARM1 overexpression improved this ability (Supplementary Fig. 2A, B). Similarly, knockout of CARM1 expression resulted in significant suppression of cellular proliferation (Supplemental Fig. 2C–E).

To further elucidate the in vivo pro-proliferative effect of CARM1, control and CARM1-knockdown Huh7 cells were subcutaneously injected into nude mice. Tumors formed by CARM1-knockdown cells exhibited significantly decelerated growth and smaller mean volumes and weights than those formed by control cells (Fig. 5D–F). Furthermore, Ki-67 is a proliferative activity marker of tumors and is often used to compare the proliferative capacity of tumors [32]. IHC staining of Ki-67 in xenografts revealed a notable reduction in the expression of Ki-67 in the CARM1-knockdown group compared to that in the control group (Fig. 5G). These results indicated that CARM1 promotes HCC proliferation in vivo and in vitro.

### CARM1 facilitates HCC metastasis

**Fig. 6:**
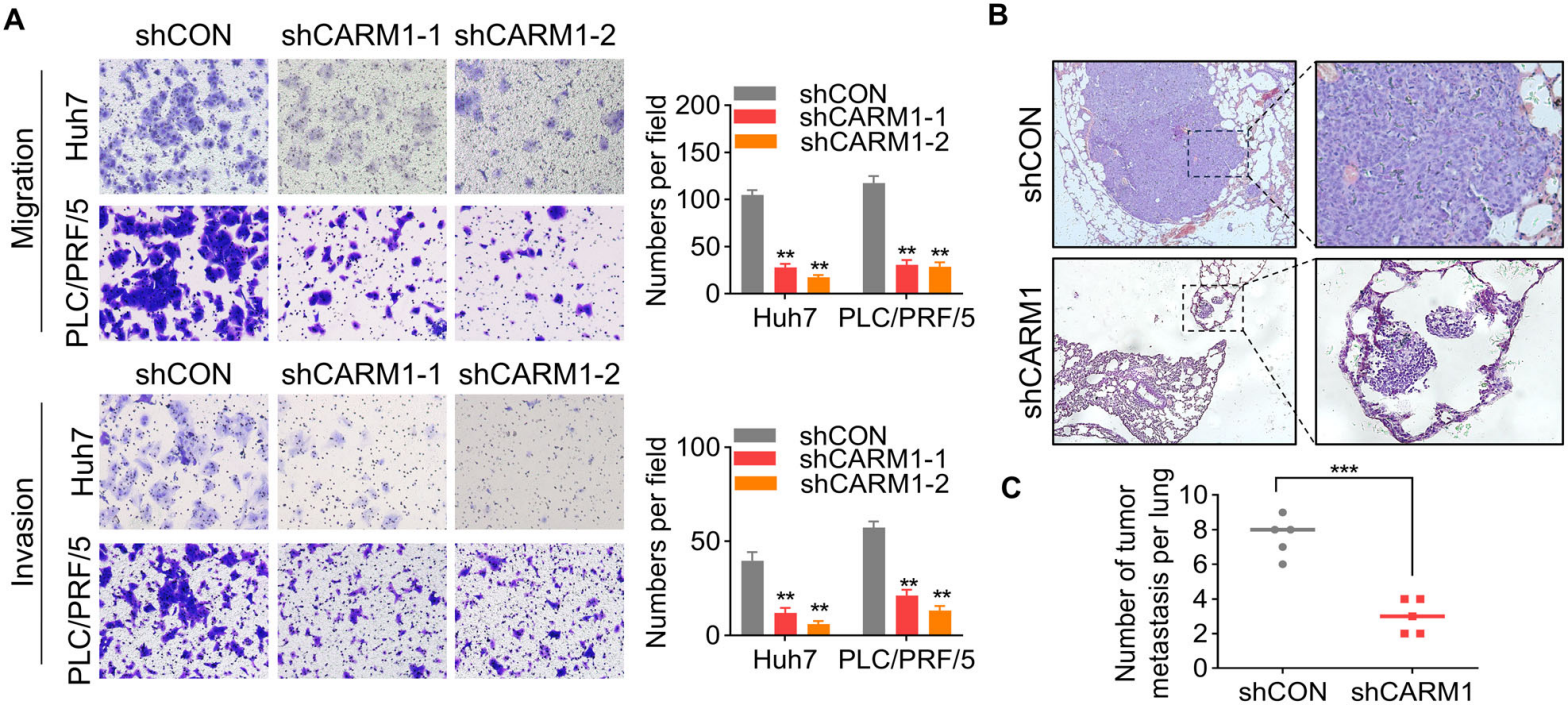
CARM1 enhances the metastatic ability of HCC cells. **A** Representative images of the transwell assay results for control and CARM1-knockdown Huh7 and PLC/PRF/5 cells showing their migration and invasion ability (left). The cells in five randomly selected fields were counted under a microscope, and the data were presented as a bar chart (right). **B** Representative microscopy images of pulmonary metastatic lesions 8 weeks after the injection of the indicated Huh7 cells into the tail vein of nude mice. **C** The number of lung metastatic tumors in each group was determined. (*n* = 3; *p < 0.05, **p < 0.01, and ***p < 0.001).

To investigate whether CARM1 influences HCC tumor metastasis, we first examined whether CARM1 affects the migratory capacity of HCC cells by conducting a transwell assay. We found that CARM1 knockdown or knockout significantly impaired the migration of HCC cells (Fig. 6A and Supplementary Fig. 2F). Similarly, cell invasion was attenuated following the knockdown or knockout of CARM1 (Fig. 6A and Supplementary Fig. 2F).

To assess the effect of CARM1 on tumor metastasis in vivo, we intravenously injected HCC cells with or without CARM1 knockdown into the tail vein of nude mice. After 2 months, the lungs were extracted, showing a remarkable decrease in the quantity of pulmonary metastatic nodules in the CARM1-knockdown group compared to the control group (Fig. 6B, C). To summarize, these findings showed that CARM1 promoted HCC cell migration and invasion in vitro, as well as tumor metastasis in vivo.

### Activation of FERMT1 transcription by CARM1-methylated H3R17 affects HCC progression

**Fig. 7:**
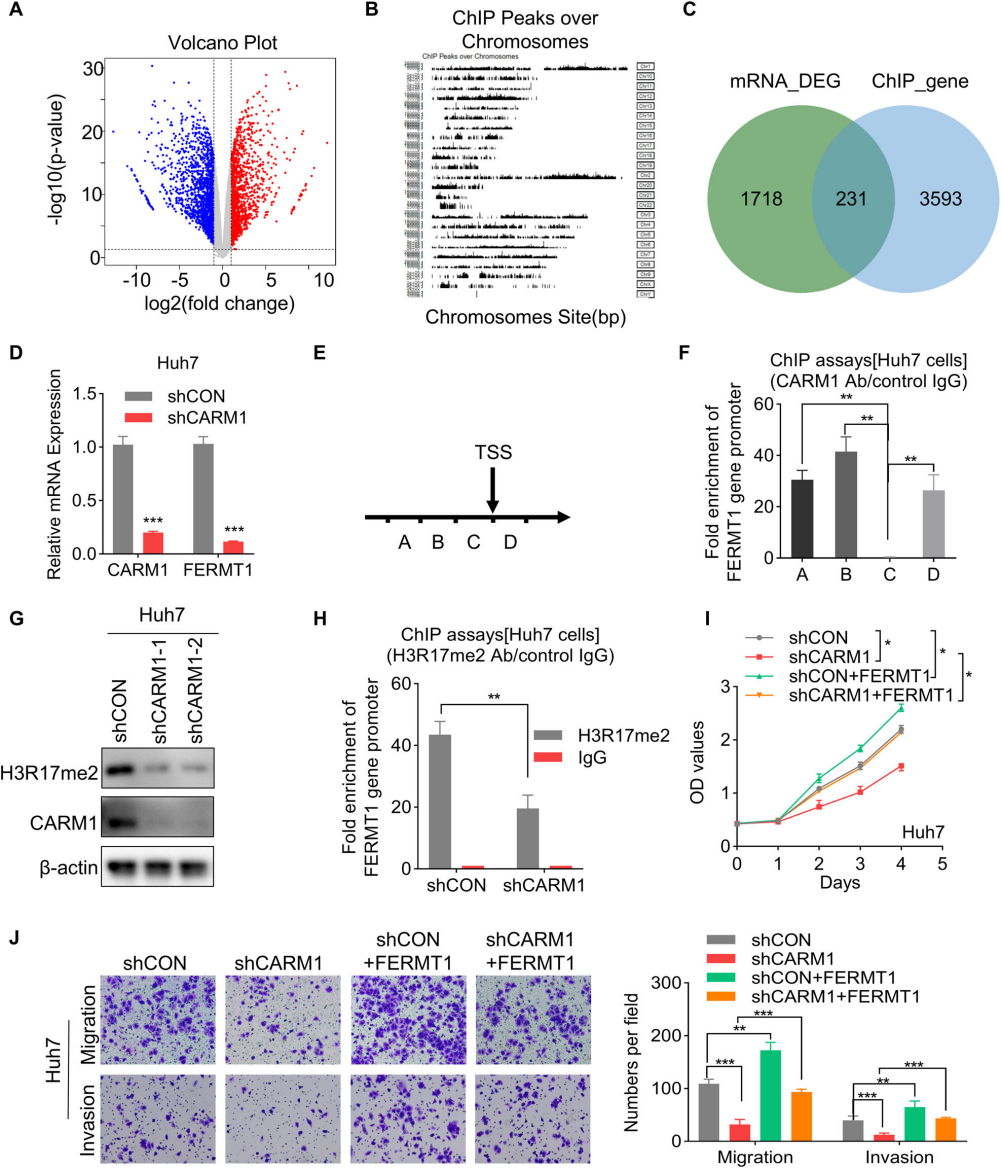
FERMT1 functions downstream of CARM1 via histone modification. **A** RNA-seq analysis revealed genes whose expression was upregulated (red) or downregulated (blue) in control and CARM1-knockdown Huh7 cells. **B** Schematic illustration of the peaks identified by ChIP-seq analysis with an anti-CARM1 antibody in Huh7 cells. **C** Integration of ChIP-seq and RNA-seq data. The Venn diagram shows the overlap between targets and differentially expressed genes. **D** Detection of the mRNA levels of CARM1 and FERMT1 in control and CARM1-knockdown Huh7 cells by qRT-PCR. **E** Schematic representation of the four segments near the TSS of FERMT1. ChIP primers were designed for each of the four sequences. **F** A ChIP assay was performed to detect CARM1 enrichment in the FERMT1 promoter region using an anti-CARM1 antibody. **G** Western blotting analysis was performed to show the expression level of H3R17me2 in control and CARM1-knockdown cells. **H** ChIP assay was performed to detect H3R17me2 enrichment in the FERMT1 promoter region using an anti-H3R17me2 antibody. The IgG antibody was used as the negative control. The inhibitory effect of CARM1 depletion on Huh7 cells, as demonstrated by rescue experiments, was effectively counteracted by the overexpression of FERMT1, as shown by both the CCK-8 (**I**) and transwell (**J**) assays. (n = 3; *p < 0.05, **p < 0.01, and ***p < 0.001).

As a histone arginine methyltransferase, CARM1 catalyzes the dimethylation of H3R17 (H3R17me2) to activate the transcription of downstream genes. However, the target genes regulated by CARM1-mediated H3R17me2 modification in HCC remain unknown. Therefore, to elucidate the mechanisms by which CARM1 regulates target genes in HCC, transcriptomic RNA sequencing combined with anti-CARM1 ChIP-sequencing (ChIP-seq) was performed (Fig. 7A, B). Integration of the transcriptome and ChIP-seq data revealed significant regulatory associations between CARM1 binding events and gene expression changes. We identified 553 differentially expressed genes that exhibited concurrent CARM1 binding within their regulatory regions, and their expression levels were closely related to CARM1 (Fig. 7C). Moreover, these 231 genes were evaluated by the Kyoto Encyclopedia of Genes and Genomes (KEGG) pathway enrichment analysis. These common genes were significantly enriched in cancer-related pathways, such as metabolism, Hippo signaling and cell adhesion molecules (Supplemental Fig. 3A). Among these genes, we selected FERM domain containing kindlin 1 (FERMT1) as a candidate target gene of CARM1 for further investigation. FERMT1 is involved in integrin signaling and the linkage of the actin cytoskeleton to the extracellular matrix, which plays an important role in enhancing tumor progression [33]. Additionally, FERMT1 was found to be involved in the regulation of the EMT pathway [34]. The expression of the FERMT1 gene was most significantly altered after knocking down CARM1, and it was found to be strongly bound by CARM1 (Supplementary Fig. 3B). TCGA analysis via the TIMER 2.0 and GEPIA2 online tools revealed a positive correlation between FERMT1 and CARM1 expression in HCC tissues (Supplemental Fig. 3C, D). We conducted a qRT-PCR assay and found that knocking down CARM1 significantly decreased FERMT1 expression (Fig. 7D). To confirm the binding of CARM1 to the FERMT1 promoter region, we performed ChIP assays using four distinct pairs of ChIP primers at the FERMT1 promoter locus (Fig. 7E). Using an antibody specifically targeting CARM1, we revealed the presence of CARM1 at the promoter region of FERMT1 in Huh7 cells. Our findings indicated that CARM1 bound to amplicons A, B, and D of FERMT1 but did not bind to amplicon C. The most proximate binding occurred within segment B (Fig. 7F).

Next, we evaluated the effect of CARM1 on H3R17me2 modification. The results revealed that H3R17me2 decreased after CARM1 was knocked down (Fig. 7G). Moreover, suppression of CARM1 expression considerably decreased H3R17me2 levels within the FERMT1 promoter, as determined by ChIP assays with an anti-H3R17me2 antibody, followed by qRT-PCR analysis (Fig. 7H). These results suggested that CARM1 activates the transcription of FERMT1 via H3R17me2.

To determine whether FERMT1 functions downstream of CARM1, rescue experiments were conducted (Supplemental Fig. 3E). The results of CCK-8 and transwell assays revealed that overexpression of FERMT1 abolished the suppression of proliferation, migration, and invasion caused by CARM1 knockdown in Huh7 cells (Fig. 7I, J). These results revealed that CARM1 activates the transcription of FERMT1 via H3R17me2, which is critical for the oncogenic role of CARM1 in the progression of HCC.

### CARM1 inhibitor restrains HCC

**Fig. 8:**
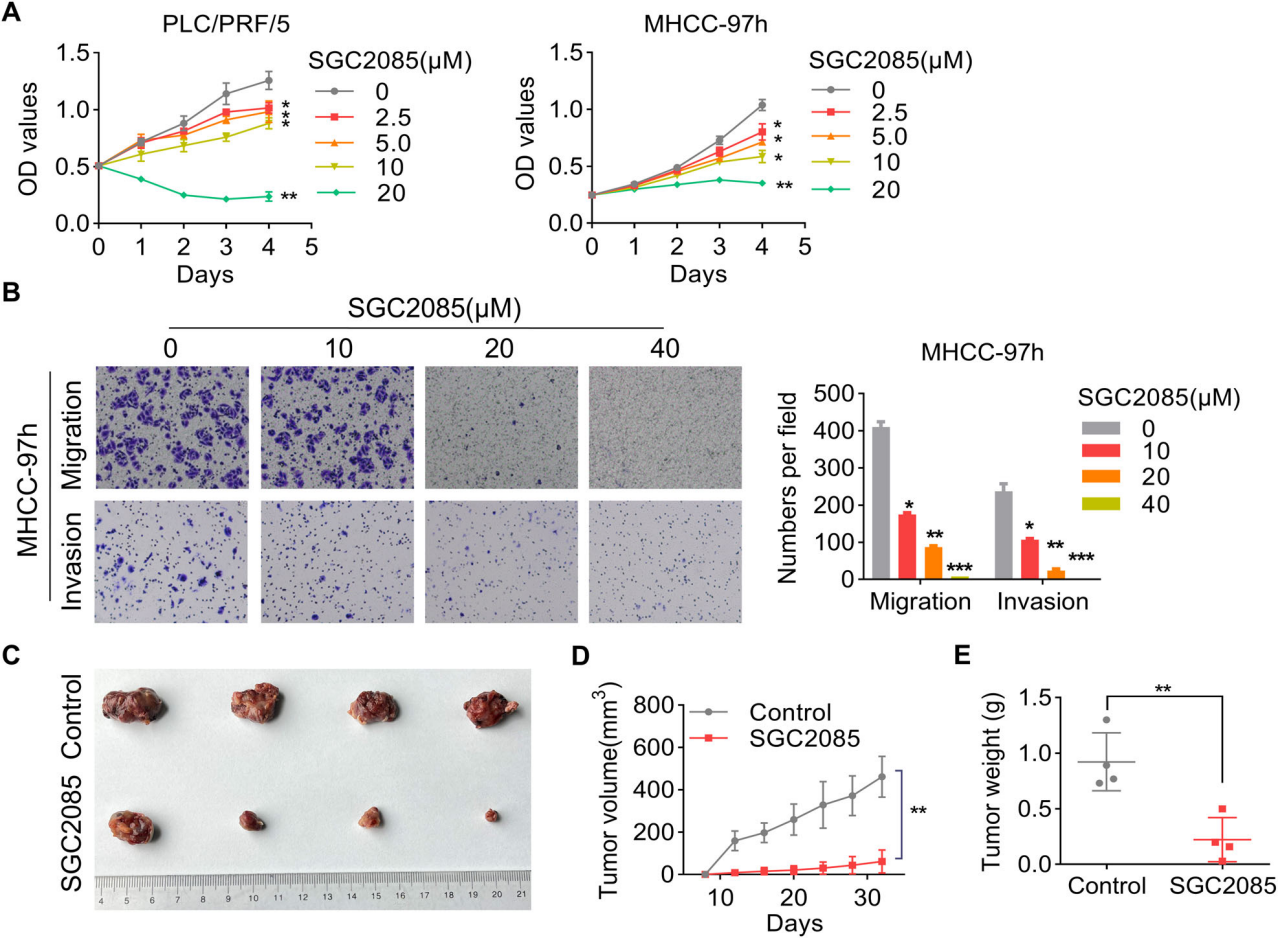
Antitumor effects of the CARM1 inhibitor SGC2085 in HCC. **A** The cytotoxicity of various concentrations of SCG2085 to MHCC-97h and PLC/PRF/5 cells was examined by a CCK-8 assay. **B** MHCC-97h cells were pretreated with the indicated concentrations of SGC2085 for 48 h. Transwell assays were conducted to evaluate the migratory and invasive potential of the cells. The accompanying figures depict representative images (left) and the corresponding statistical findings derived from the transwell assay (right). Nude mice with subcutaneous tumors formed by MHCC-97h cells were treated with vehicle control or SGC2085 (10 mg/kg; intraperitoneally (i.p.), three times each week) for 24 days (**C**). The tumor volume was measured every 3 days, and the data were presented as a line graph (**D**). The tumor weights of the xenografts from the different groups were calculated (**E**). (*n* = 3; *p < 0.05, **p < 0.01, and ***p < 0.001).

A study showed that SGC2085, a potent and selective inhibitor, can inhibit CARM1 activity [35]. Based on our findings that CARM1 functions as an oncogene in HCC, we speculated that SGC2085 might have an inhibitory effect on HCC cells. To test our hypothesis, HCC cells were exposed to different concentrations of SGC2085. Notably, the administration of SGC2085 considerably inhibited proliferation, migration, and invasion in a dose-dependent manner (Fig. 8A, B). Moreover, administering SGC2085 substantially suppressed tumor growth in vivo, as confirmed by the outcomes of the xenograft model (Fig. 8C–E). To summarize, these findings supported the therapeutic potential of the CARM1 inhibitor SGC2085 on HCC.

## Discussion

HCC is the predominant form of primary liver cancer, constituting about 90% of cases. With 910,000 newly diagnosed cases and 830,000 deaths, it is the sixth most prevalent cancer and the third leading cause of mortality [36]. Surgical resection, liver transplantation, radiotherapy, chemotherapy, and molecular targeted therapy have been widely used as primary approaches in the clinical management of HCC. However, owing to its undetectable onset, early symptoms and signs of HCC often remain hidden. By the time symptoms manifest and patients seek medical attention, the disease often progresses to an advanced stage, depriving them of the opportunity for curative surgery. Moreover, postoperative recurrence and metastasis significantly affect the treatment outcomes and overall survival of HCC patients [37]. Therefore, understanding the underlying molecular mechanisms of HCC occurrence and progression can help identify effective biomarkers for early diagnosis and prognosis prediction, as well as discover novel drug targets to overcome therapeutic barriers in the treatment of HCC. By analyzing our cohort and TCGA database, we revealed a significant increase in CARM1 expression in HCC tissue samples. Moreover, the correlation analysis between CARM1 expression and clinicopathological features revealed that CARM1 overexpression was strongly associated with the presence of satellite foci, increased serum HBV and AFP levels, and a poor prognosis. Consistent with our findings, a recent investigation revealed that CARM1 expression is notably correlated with survival and various clinical parameters, including alpha-fetoprotein levels, tumor size, satellite nodules, and microvascular invasion [38]. These findings provided additional support for the potential of CARM1 as a prognostic indicator in patients with HCC.

Post-translational modifications, including phosphorylation, O-acetylglycosylation, and methylation, regulate CARM1 function [15–18]. Several studies have reported the critical role of ubiquitination in modulating CARM1 stability. For example, the E3 ligase S-phase kinase-associated protein 2 (SKP2) interacts with CARM1 to facilitate the ubiquitin-mediated degradation of CARM1 [19]. Tripartite motif-containing protein 28 (TRIM28) physically interacts with CARM1, decreasing its ubiquitination and protecting it from proteasome-mediated degradation [38]. However, whether deubiquitinase is responsible for regulating CARM1 stability is still unclear. The PSMD14 protein plays a crucial role in various biological processes, including DNA damage repair, cellular differentiation, and multidrug resistance [24, 39, 40]. Moreover, abnormal overexpression of PSMD14 contributes to tumor growth and metastasis. PSMD14 increases the stability of the Snail protein, a transcription factor associated with epithelial-mesenchymal transition (EMT), thus inducing metastasis in esophageal cancer cells [41]. In HCC, PSMD14 stabilizes E2F1, which upregulates survivin and FOXM1 expression, thus facilitating the growth of HCC [26]. PSMD14 also influences HCC metastasis by deubiquitinating the TGF-β receptor and caveolin-1 [28]. Our previous study revealed that GRB2 is a target of PSMD14-mediated deubiquitination, which is critical for the progression of HCC. In this study, we identified CARM1 as a novel substrate of PSMD14. CARM1 was important for PSMD14-enhanced proliferation, migration, and invasion in HCC cells. The interaction between PSMD14 and CARM1 led to a reduction in CARM1 ubiquitination and protected it from degradation. Notably, residue K363 of CARM1 exclusively plays a crucial role in this process. We also found that PSMD14 efficiently deubiquitinates CARM1 by removing the K48-linked and K63-linked polyubiquitin chains, and this finding was similar to previous studies, revealing that PSMD14 is responsible for removing the K48-linked and K63-linked ubiquitin chains of target proteins [23, 42]. K48-linked and K63-linked polyubiquitin chains act as discerning signs of the proteasome, enabling efficient processing of substrates within its confines [42]. Our investigation also revealed a synchronized increase in the expression of PSMD14 and CARM1 in clinical HCC samples and its significance for the prognosis of patients, suggesting strong pathological implications for the regulation of CARM1 by PSMD14 in the progression of HCC.

Along with its involvement in transcription regulation, pre-mRNA splicing, and the cell cycle, CARM1 plays various roles in different cellular processes, including DNA end resection, the replication stress response, cell autophagy, tumor immunity, and tumor metabolism [7, 11, 43–45]. In HCC, CARM1 promotes malignant behaviors by activating the AKT/mTOR signaling pathway [38]. This study revealed that CARM1 functions as an oncogene to facilitate HCC growth and metastasis. However, another study revealed that glucose starvation-induced CARM1 expression induces GAPDH hypermethylation to suppress glycolysis in liver cancer cells, indicating a tumor-suppressive role of CARM1 [46]. The contradictory effects of CARM1 may arise from the distinct metabolic environments of tumor cells, potentially involving different primary pathways of influence.

FERMT1 is a novel adhesion protein belonging to the kindlin family and consists of ezrin, radisin, moesin, and a pleckstrin homology domain. It is a mutant gene closely associated with Kindler syndrome [47]. It plays an important role in regulating cell cycle arrest, apoptosis, EMT, motility, invasiveness, and angiogenesis in cancer. FERMT1 activates the NF-κB, Wnt/β-catenin, and PI3K/AKT signaling pathways and is associated with Nod-like receptor family protein 3 [48–51]. Some small RNAs, such as hsa_circ_0000554 and miR-24, can regulate FERMT1 expression [52, 53]. Here, we identified FERMT1 as a downstream gene of CARM1 and found that FERMT1 is important for CARM1-induced malignant behaviors. Although CARM1 can methylate both H3R17 and H3R26, it preferentially methylates H3R17 [54]. Therefore, we estimated H3R17me2 levels at the FERMT1 promoter and found that CARM1 knockdown significantly decreased H3R17me2 levels. CARM1 forms transcriptional complexes with PAF1, AR, ER, or P300 to activate downstream gene expression [8–10]. Further investigations are needed to identify the specific transcription factor involved in the CARM1-mediated activation of FERMT1 transcription.

As CARM1 is overexpressed and functions as an oncogene in various types of tumors, targeting CARM1 may help in cancer treatment. For example, the CARM1-specific inhibitors TP-064 and EZM2302 have demonstrated their effectiveness as promising therapeutic interventions for multiple myeloma [55, 56]. In this study, we found that administering SGC2085 markedly inhibited the proliferation, migration, and invasion of HCC cells in vitro and strongly suppressed tumor growth in vivo, suggesting that targeting CARM1 may be a promising treatment for HCC. In the last five years, immune checkpoint inhibitors have revolutionized the management of HCC. The inhibition of CARM1 induces high antitumor efficacy in cytotoxic T-cells and tumor cells [45]. The combination of the CARM1 inhibitor EZM2302 and an anti-PD-1 antibody exerted promising synergistic effects on a preclinical model of lung cancer [57]. These findings suggested that the combination of CARM1 inhibitors and immunotherapy may generate synergistic effects in the treatment of HCC.

## Conclusion

**Fig. 9:**
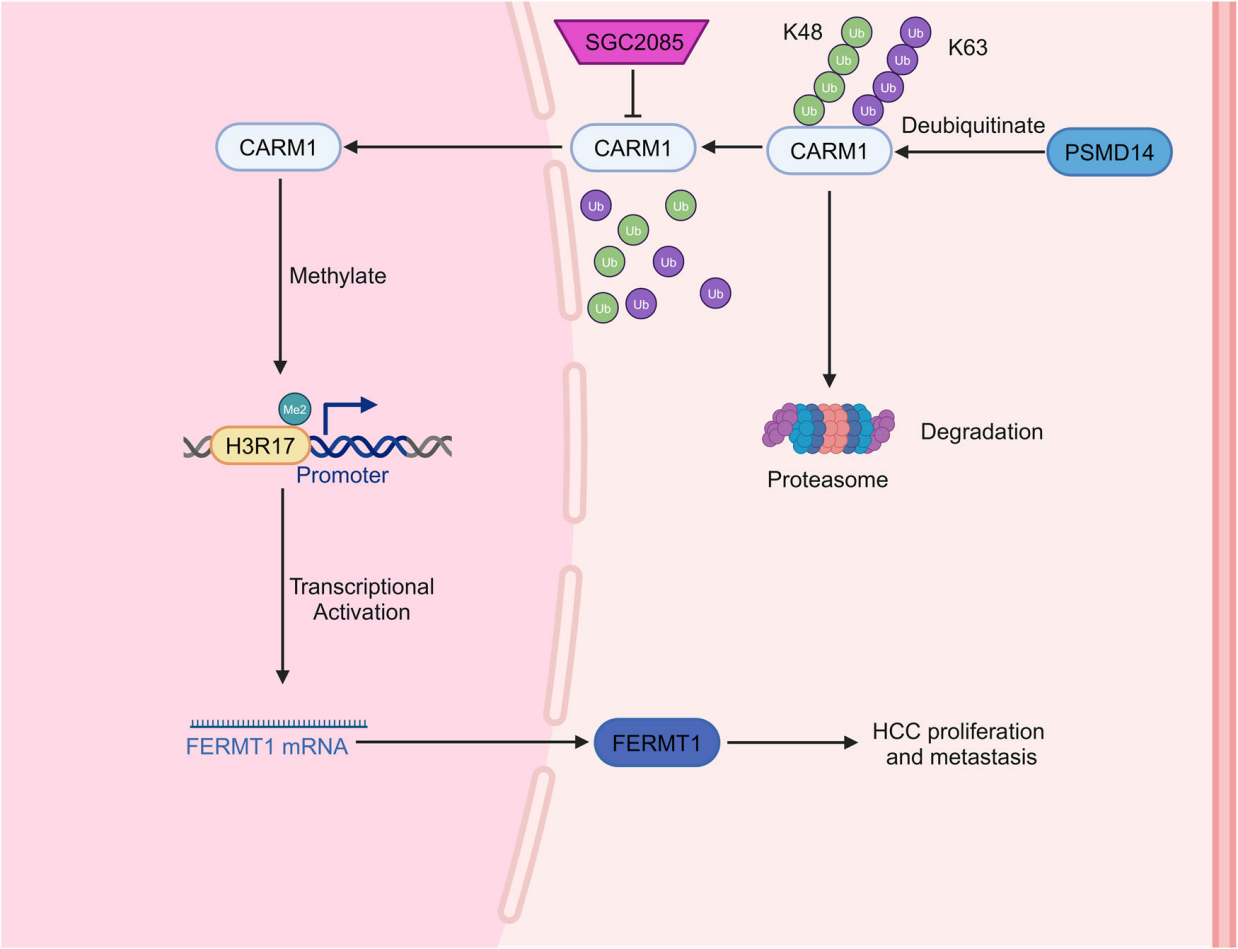
Schematic diagram of the PSMD14-CARM1-FERMT1 axis in HCC. PSMD14-mediated deubiquitination upregulates CARM1 expression, which in turn transcriptionally activates its downstream target FERMT1 through histone H3R17me2. This PSMD14-CARM1-FERMT1 signaling axis significantly promotes HCC growth and metastasis. Pharmacological inhibition of CARM1 using SGC2085 effectively suppresses the malignant phenotypes of HCC cells, suggesting a potential therapeutic strategy for HCC treatment.

To summarize, our findings revealed that CARM1 is stabilized by PSMD14-mediated deubiquitination and activates FERMT1 transcription through H3R17me2 modification (Fig. 9), indicating a key role in the progression of HCC. Thus, directing therapeutic efforts toward CARM1 may be a promising strategy to treat HCC.

## Supplementary information

Supplementary Figure legends

Supplementary Figure 1

Supplementary Figure 2

Supplementary Figure 3

Original images of Western blotting

## Data Availability

The data substantiating the findings of this study can be obtained from the corresponding authors upon reasonable inquiry.
