# Supplementary material for: PSMD14-mediated deubiquitination of CARM1 facilitates the proliferation and metastasis of hepatocellular carcinoma by inducing the transcriptional activation of FERMT1
Source: Cell Death Dis. 2025 Feb 27;16(1):141. doi: 10.1038/s41419-025-07416-3 (PMC11868421; doi:10.1038/s41419-025-07416-3)
Supplement: Supplementary file 1 — Supplementary Figure legends [file 41419_2025_7416_MOESM1_ESM.docx]

**Supplemental Figure legends**

**Supplemental Figure 1. CARM1 is upregulated in HCC.**

A. Differential expression of CARM1 between tumor and adjacent normal tissues across all TCGA tumors.

B. Expression of CARM1 mRNA in HCC based on individual cancer stage in the TCGA database.

C. Expression of CARM1 mRNA in HCC based on tumor grade in the TCGA database.

(*p < 0.05, **p < 0.01, and ***p < 0.001).

**Supplemental Figure 2. The oncogenic role of CARM1.**

A. Proliferation of control and CARM1-overexpressing PLC/PRF/5 and MHCC-97h cells was detected by CCK-8 assays on the indicated days.

B. A colony formation assay (left) was performed to detect the proliferation of control and CARM1-overexpressing PLC/PRF/5 and MHCC-97 h cells. The data are presented as a bar chart (right).

C. The CARM1 was knocked out using sgRNAs in Huh7 and PLC/PRF/5 cells.

D. The proliferation of control and CARM1-knockout Huh7 and PLC/PRF/5 cells was tested using CCK-8 assay.

E. The colony formation of control and CARM1-knockout Huh7 and PLC/PRF/5 cells.

F. The migration and invasion of control and CARM1-knockout Huh7 and PLC/PRF/5 cells was tested using transwell assay.

(n = 3; *p < 0.05, **p < 0.01, and ***p < 0.001).

**Supplemental Figure 3. CARM1 activates FERMT1 transcription.**

A. The Kyoto Encyclopedia of Genes and Genomes (KEGG) pathway enrichment analysis of common genes from RNA-seq and ChIP-seq.

B. Peak distribution of FERMT1 was determined by ChIP-seq. The input was taken as the background, Macs2 was used to call peaks on the IP, and the narrow peak mode was used for peak calling. Macs2 was used to predict the length of the protein-binding sequence through modeling and to determine the relative abundance of the peak corresponding to the sequence through the length of the sequence and the number of paired-end reads compared to the sequence.

C-D. Gene expression correlation of CARM1 and FERMT1 in the TCGA cohort was determined using TIMER 2.0 (C) and GEPIA2 (D) separately.

E. Myc-tagged FERMT1 was transfected into Huh7 cells in which CARM1 was knocked down. Then, a Western blotting assay was performed.
