# Supplementary figures and images for: PSMD14-mediated deubiquitination of CARM1 facilitates the proliferation and metastasis of hepatocellular carcinoma by inducing the transcriptional activation of FERMT1
Source: Cell Death Dis. 2025 Feb 27;16(1):141. doi: 10.1038/s41419-025-07416-3 (PMC11868421; doi:10.1038/s41419-025-07416-3)

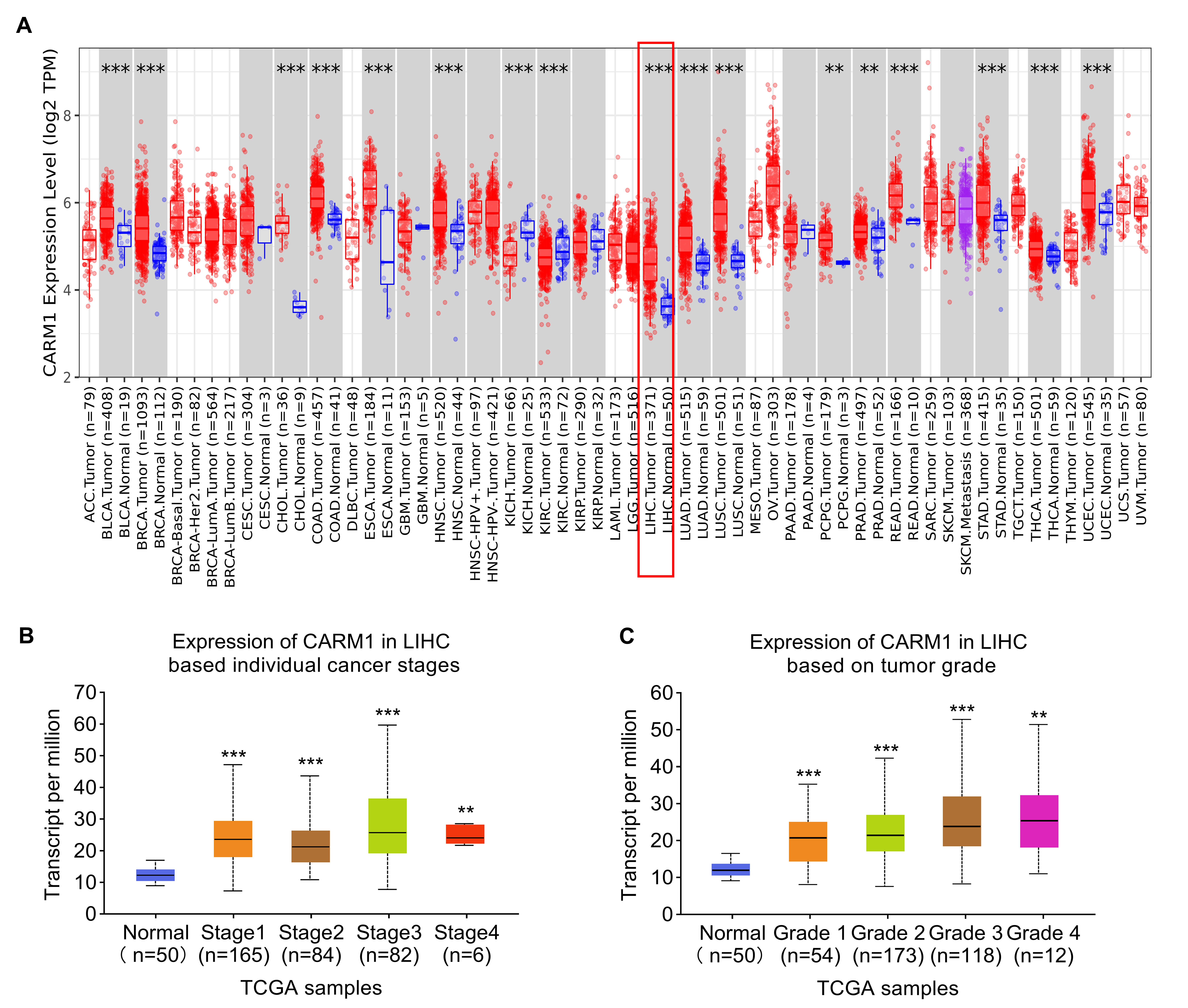

Supplement: Supplementary file 2 — Supplementary Figure 1 [file 41419_2025_7416_MOESM2_ESM.tif]

Fig. 1A

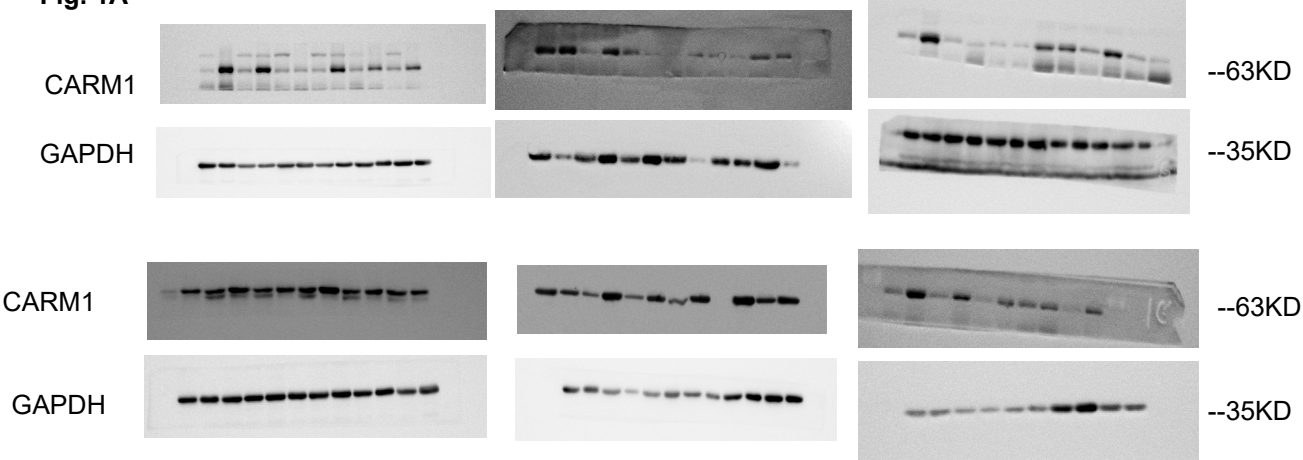

**Fig. 2B**

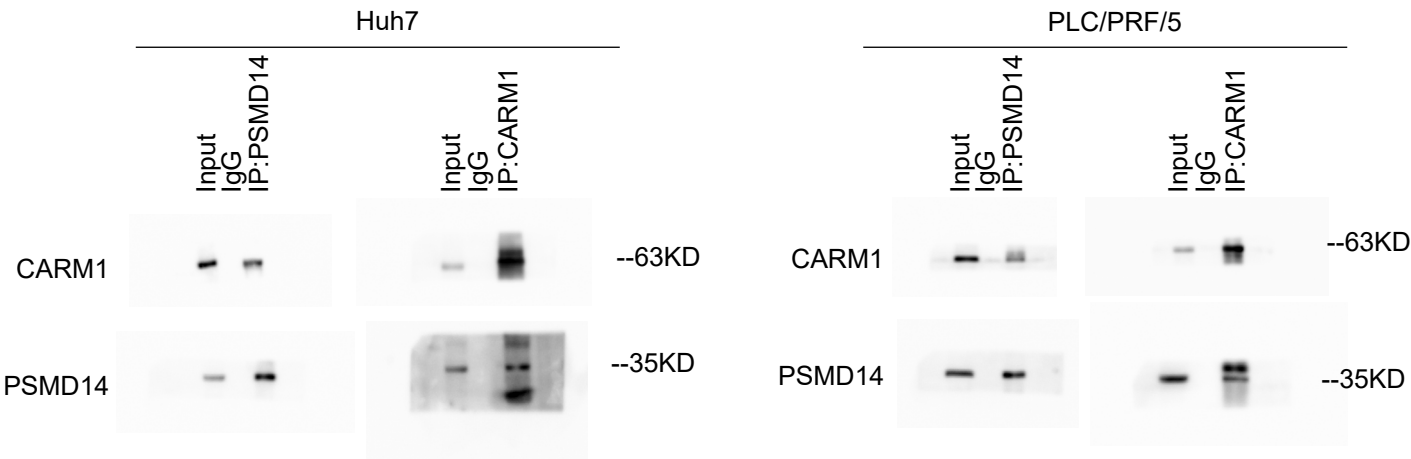

**Fig. 2C**

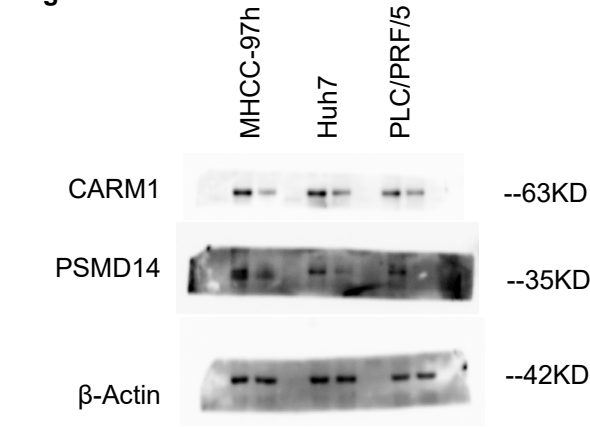

**Fig. 2E**

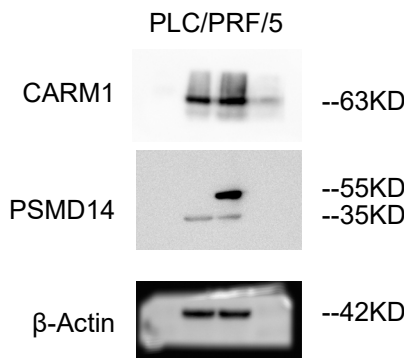

**Fig. 2G**

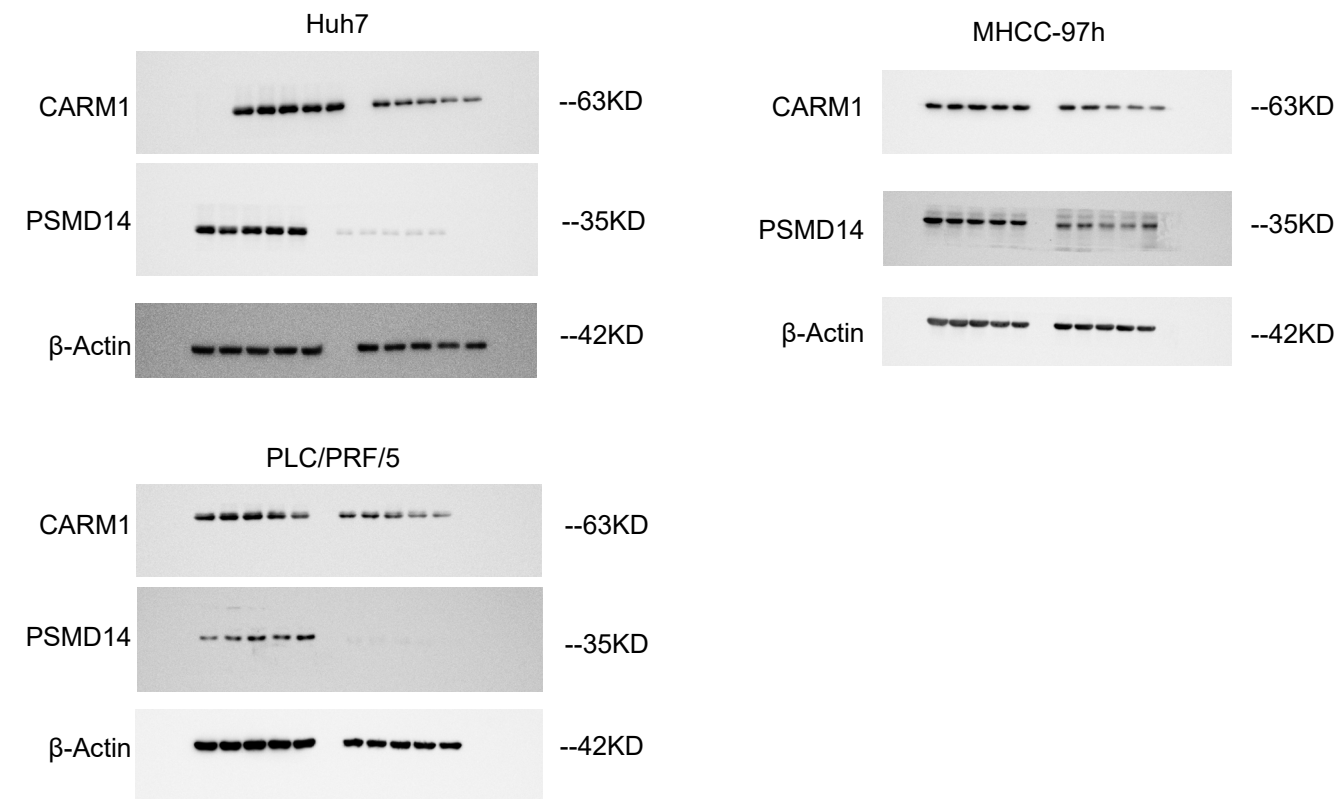

**Fig. 3A**

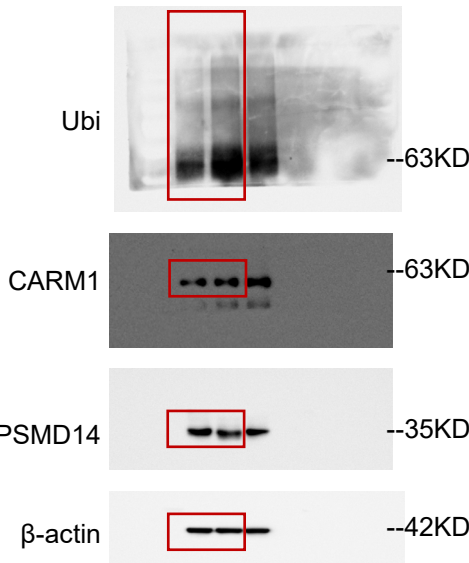

**Fig. 3B**

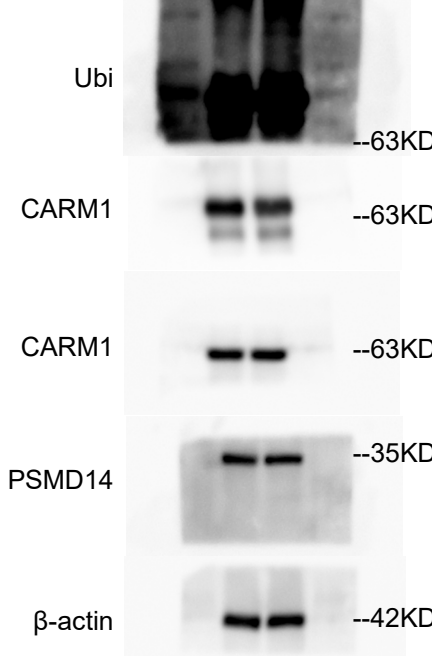

**Fig. 3C**

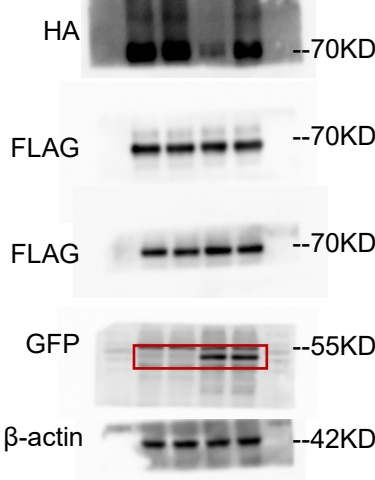

**Fig. 3D**

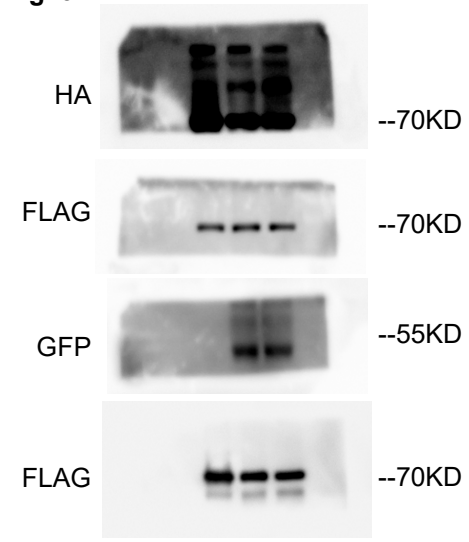

**Fig. 3E**

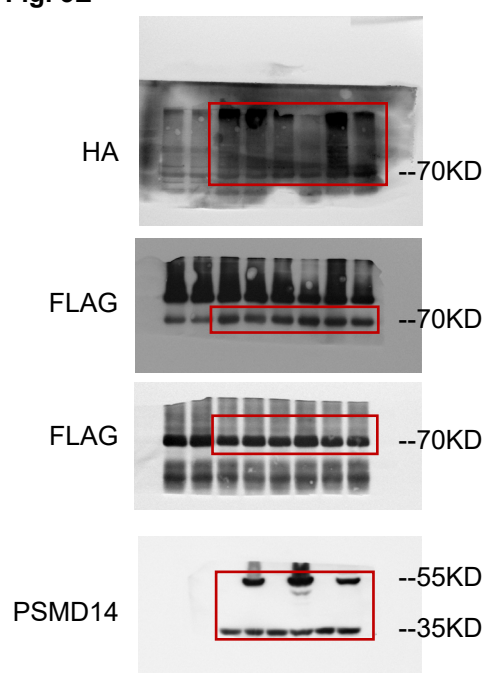

**Fig. 3G**

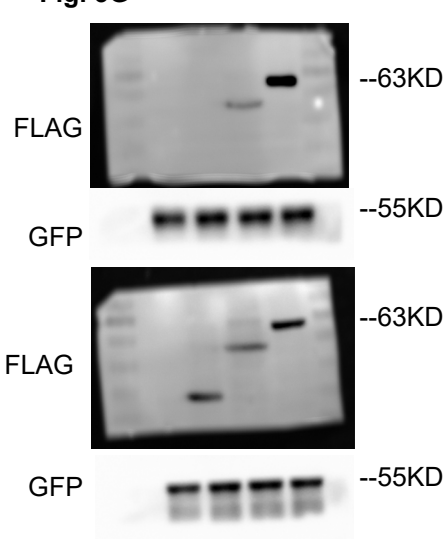

**Fig. 3H**

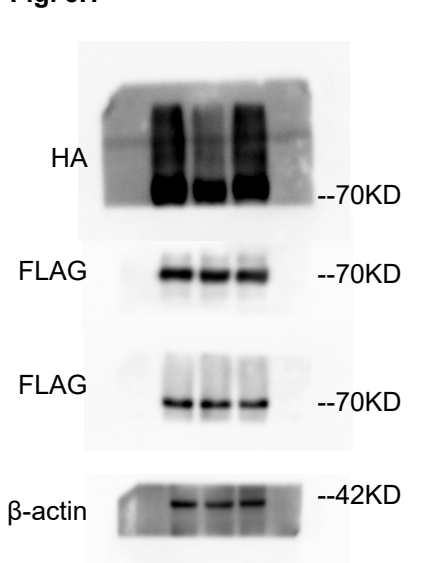

**Fig. 5A**

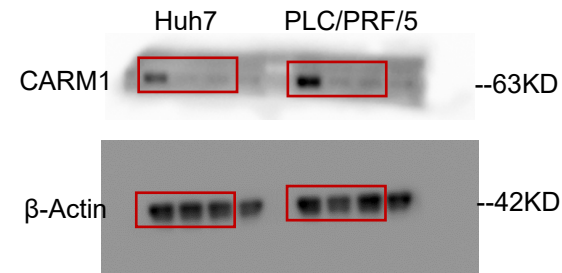

**Fig. 7G**

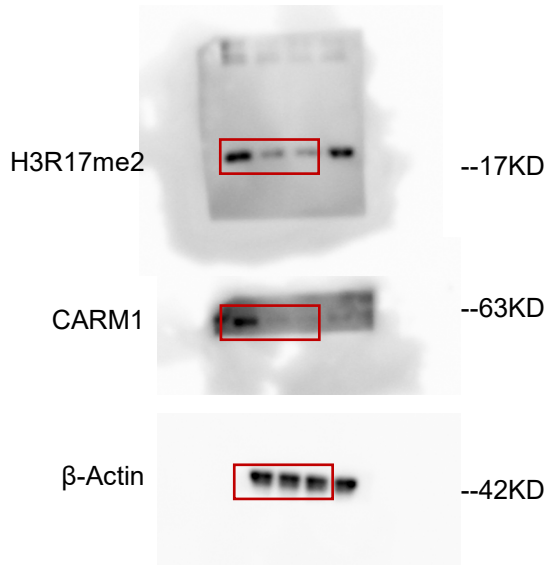

**Sup Fig. 2C**

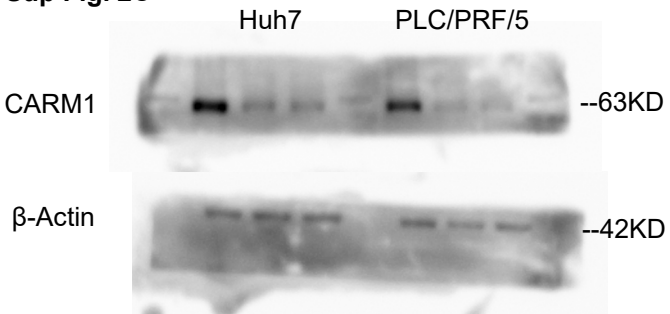

**Sup Fig. 3E**

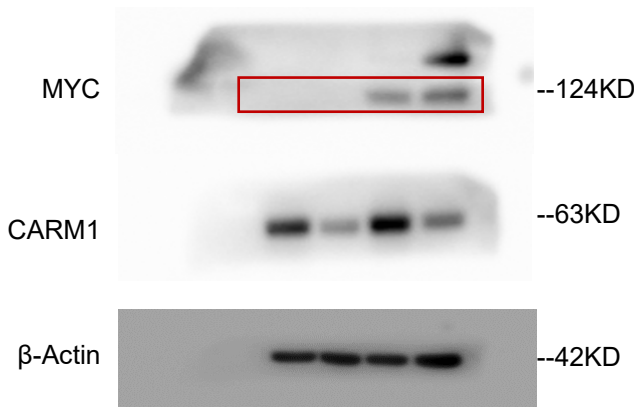

Supplement: Supplementary file 5 — Original images of Western blotting [file 41419_2025_7416_MOESM5_ESM.pdf]
